# Foliar application of nano-NPK and calcium-boron during the ‘off-year’ season enhances yield, improves oil quality, and mitigates biennial bearing in ‘Picual’ olive cultivar

**DOI:** 10.1186/s12870-026-08984-y

**Published:** 2026-05-25

**Authors:** Ayman Shaban, Hassan Mahmoud Korkar, Islam Ahmed, Asmaa Gamal Abd El-hamied, Ibrahim Hmmam

**Affiliations:** 1 Pomology Department, Faculty of Agriculture, Cairo University, PO Box 12613, Giza, Egypt; 2 The Higher Institute for Agricultural Co-Operation, Cairo, Egypt; 3 Food Technology Institute, Agricultural Research Center, Ministry of Agriculture, Giza, Egypt

**Keywords:** Alternate bearing index, Calcium-boron, Flowering, Fruit set, NPK, *Olea europaea* L., Olive oil quality, Picual cultivar

## Abstract

**Background:**

Global olive cultivation is often hindered by the physiological phenomenon known as alternate bearing, which results in inconsistent yields from year to year, thereby diminishing economic returns and challenging effective orchard management. While foliar nutrition is acknowledged as a viable approach to boost reproductive performance in olive trees, there is limited research exploring the combined use of NPK and calcium boron nano fertilizers (NPK-NF and CaB-NF) during the ‘off-year’ season to concurrently enhance flowering, fruit set, oil composition, and reduce the severity of alternate bearing.

**Results:**

Field experiments conducted during the 2023 and 2024 seasons on fifteen-year-old ‘Picual’ olive trees demonstrated that foliar application of NPK-NF and CaB-NF significantly enhanced reproductive performance and oil quality while reducing alternate bearing severity. Trees receiving NPK-NF (4 mL/L) + CaB-NF (2 mL/L) exhibited maximum flowering parameters, including 9.31 inflorescences per shoot and 68.51% perfect flowers, alongside the highest initial fruit set (37.37%) and final fruit set (26.95%). This treatment produced the greatest ‘off-year’ yield (0.79 tonnes per acre) while reducing fruit drop to 27.83% compared to 51.77% in controls. Leaf and fruit mineral analysis revealed substantially enhanced nitrogen, phosphorus, potassium, and calcium concentrations in treated trees. Virgin olive oil maintained extra virgin classification with improved oleic acid proportions, reaching 67.85%, and increased pigment concentrations. Notably, the combination of NPK-NF (4 mL/L) + CaB-NF (3 mL/L) reduced alternate bearing severity to 31.06% compared to 88.08% in control trees.

**Conclusion:**

These findings suggest that the deliberate foliar application of NPK-NF and CaB-NF during the ‘off-year’ season can significantly boost flowering, fruit set, and oil quality, while also alleviating the severity of alternate bearing in ‘Picual’ olive cultivar. This approach offers a viable nutritional strategy to enhance both yield stability and productivity in commercial olive orchards.

**Supplementary Information:**

The online version contains supplementary material available at 10.1186/s12870-026-08984-y.

## Introduction

The olive tree (*Olea europaea* L.), a member of the Oleaceae family, is an evergreen species believed to be one of the oldest cultivated trees. Several theories suggest that its original region includes the Eastern Mediterranean Basin, Anatolia, Syria and Central Mesopotamia [1]. The Mediterranean region remains the center of cultivation, producing about 99% of the world’s olive oil and consuming about 87% of it [2].

Its physiological adaptations to drought and salinity, such as low stomatal conductance and osmotic adjustment, make it suitable for arid and semi-arid environments [3]. This resilience underlines its vital economic importance throughout the Mediterranean. The fruit is mainly processed to extract oil or for pickling as table olives, both products valued for their nutritional richness in antioxidants and beneficial fatty acids [4]. Globally, the olive harvest area reached approximately 11 million hectares in 2024, with a production of approximately 22.9 million tonnes [5]. In Egypt, olive cultivation continues to play a significant role in the agricultural sector. In 2024, the harvested area reached 117.744 hectares, with an average yield of 10.784.4 kg/ha, resulting in a total production of approximately 1.269.795 tonnes [5].

A principal challenge in olive production is alternate bearing, the pronounced cycle of high-yield (‘on-year’) and low-yield (‘off-year’) seasons [6–8]. Regulating flowering and fruit set, which are highly sensitive to nutritional status, is therefore a key management objective. Adequate nitrogen (N) is crucial for flower formation and fruit set [7, 9, 10], while phosphorus (P) supports energy metabolism and cell division [11]. Potassium (K) is vital for carbohydrate transport and osmoregulation [12]. Calcium (Ca) ensures cell wall integrity [13], and the micronutrient boron (B) is decisive for pollination and fertilization [14]. This nutritional balance directly influences the quality of virgin olive oil, a product defined by mechanical extraction [15] and shaped by agronomic and environmental factors [16–18].

Conventional foliar fertilizers frequently exhibit limited efficacy, as their particle dimensions often exceed the 100-nanometer threshold critical for optimal leaf penetration [19]. In contrast, nano-fertilizers, characterized by particle sizes typically below 100 nanometers, offer a compelling alternative. The elevated surface area inherent to these nanomaterials facilitates enhanced interfacial interactions with foliar tissues, thereby improving nutrient use efficiency [19–22]. Investigations into nano-fertilizer applications have spanned diverse fruit crops, including citrus [23], mango [24, 25], and date palm [26, 27]. Although preliminary studies on olive trees have been investigated [22, 28–30], there remains a conspicuous absence of comprehensive research addressing flowering physiology, alternate bearing tendencies, and oil quality parameters, particularly for economically significant cultivars such as ‘Picual’. Our earlier study conducted by Hmmam et al. [22] examined the foliar application of NPK nano-fertilizers during an ‘on-year’ season, a phase typically associated with elevated fruiting intensity. The investigation subsequently evaluated the persistence of these treatments by assessing their influence on the same trees in the following ‘off-year’. In contrast, the present work commenced nutrient applications at the beginning of an off-year cycle, utilizing foliar formulations of NPK-NF in combination with CaB-NF. Tree performance during that season was assessed through measurements of flowering dynamics, yield parameters, and oil quality attributes. Furthermore, the treated trees were followed into the subsequent production cycle, and their productivity was documented during the next high-bearing ‘on-year’ season.

Hence, the present study investigates the foliar application of NPK and CaB nano-fertilizers (NPK-NF and CaB-NF) on ‘Picual’ olive trees during the ‘off-year’ season. The primary objectives are to evaluate their efficacy in promoting flowering and fruit set, improving virgin olive oil quality, and reducing the severity of alternate bearing.

## Materials and methods

The olive experimental orchard of the Higher Institute for Agricultural Cooperation, located on the Cairo-Alexandria Desert Road in Egypt (30°10’15.0"N 30°44’47.0"E), was the site of this study, conducted with full institutional permission over the 2023 and 2024 growing seasons. Meteorological data, including average day and night temperatures (°C) ranges, average humidity (%), and wind speed (km/h) for both seasons, are summarized in Supplementary Data S1. Healthy, fifteen-year-old ‘Picual’ (*Olea europaea* L.) olive trees were used in this experiment. The trees were planted in sandy soil at a spacing of 6 × 6 m and irrigated using a drip system. Irrigation water used throughout the experiment was analyzed for chemical properties. The water had a pH of 7.64, electrical conductivity of 7.01 dS/m, total dissolved solids of 4486 mg/L, and a sodium adsorption ratio of 11.70%. The detailed ionic composition is reported in our previous study [22]. For each experimental season, thirty cultivated ‘Picual’ olive trees were selected. The ‘Picual’ olive cultivar was identified by Prof. Dr. Ayman Shaban, Department of Pomology, Cairo University, Egypt. Based on previous productivity data, all selected trees for each season were in an ‘off-year’ of the alternate bearing cycle, uniform in size, shape, and productivity, and had been subject to the same horticultural practices. During the first season, a group of trees in their ‘off-year’ season was selected for evaluation under the applied treatments and subsequently monitored for yield in the following ‘on-year’ season. In the second experimental season, a different group of trees, also in their ‘off-year’ season, was selected and evaluated in the same manner, with yield assessed during the subsequent ‘on-year’ season. Details of the experimental procedures on a monthly basis are given in Supplementary Data S2. The selected trees were treated with NPK nano-fertilizers (NPK-NF) in addition to CaB nano-fertilizers (CaB-NF). The NPK and CaB nano-fertilizers were sourced from nanotech company (http://www.nanotecheg.com/) located in Dreamland, Wahat road, 6^th^ of October City, Giza, Egypt, and were used according to the manufacturer’s instructions. The final NPK-NF product had a grade of N19%, P19%, and K19%, while the final CaB-NF product had a grade of Ca10% and B0.5%. The size and morphology of the synthesized NPK-NF were characterized by the manufacturer using transmission electron microscopy (TEM) (JEOL JEM-2100, Tokyo, Japan), as previously reported by Hmmam et al. [22]. Similarly, the size and morphology of the synthesized CaB-NF were characterized by the manufacturer using TEM (JEOL JEM-2100, Tokyo, Japan). Further nanoscale characterizations of both products were performed using Energy Dispersive X-ray (EDX) spectroscopy and FTIR spectroscopy by Nawah Scientific (https://nawah-scientific.com/).

The application rates for both fertilizers were 2, 3, and 4 mL of the respective final obtained nano product per liter of water. The treatment structure was as follows:

- T0: Control (untreated)
- T1: NPK-NF (2 mL/L) + CaB-NF (2 mL/L)
- T2: NPK-NF (2 mL/L) + CaB-NF (3 mL/L)
- T3: NPK-NF (2 mL/L) + CaB-NF (4 mL/L)
- T4: NPK-NF (3 mL/L) + CaB-NF (2 mL/L)
- T5: NPK-NF (3 mL/L) + CaB-NF (3 mL/L)
- T6: NPK-NF (3 mL/L) + CaB-NF (4 mL/L)
- T7: NPK-NF (4 mL/L) + CaB-NF (2 mL/L)
- T8: NPK-NF (4 mL/L) + CaB-NF (3 mL/L)
- T9: NPK-NF (4 mL/L) + CaB-NF (4 mL/L)

To assess their impact on flowering and fruit set during the ‘off-year’ season (typically spanning April to October), NPK-NF and CaB-NF were applied as foliar sprays at three specific times: the first week of December, the first week of January, and the first week of February. An average spray volume of 18 L was applied per tree during each season. Spraying was carried out early in the morning, just after sunrise. In each treatment, the CaB-NF solution was sprayed immediately after the application of the NPK-NF to ensure sequential nutrient delivery and minimize the risk of antagonistic interactions prior to foliar uptake.

### Measurements during the ‘off-year’ season

In December of each season (December 2022 for the 2023 season; and December 2023 for the 2024 season), prior to the application of nano‑fertilizers, 28 healthy, unbranched, shoots intended for fruit production in the coming season were randomly selected and tagged on each replicate tree. To ensure uniform distribution, seven shoots were chosen from each side of the tree. This resulted in 84 shoots per treatment. To enable repetition of the experiment under identical selection parameters, a new group of trees was assigned in the second ‘off-year’ season. As noted earlier, tree selection was based on historical productivity records. The following measurements were then recorded on the selected shoots:

#### Flowering parameters

For each shoot, the total number of inflorescences was recorded, and the mean number per shoot was calculated. Flowering density was calculated using the Equation 1:

1 $$\begin{aligned} \text{Flowering density} = \:&[\text{Number of inflorescences per shoot} \\&/ \text{Shoot length (m)}] \end{aligned}$$

To estimate the perfect flowers percentage and the average number of flowers per inflorescence, 30 inflorescences per replicate were selected at random. The average number of flowers in each inflorescence was counted, and the percentage of perfect flowers was computed using the Equation 2 given by Fouad et al. [31]:

2 $$\begin{aligned} &\text{Perfect flowers}\:(\%) = [\text{Number of perfect flowers} \\& / \text{Number of total flowers per inflorescence}]\times{100} \end{aligned}$$

#### Fruiting parameters

Initial and final fruit set in addition to fruit drop were evaluated based on standard formulas. Initial fruit set % was calculated after three weeks of flowering (about 20 days of full bloom) using the Equation 3 established by Fernandez and Gomez [32]:

3 $$\begin{aligned} &\text{Initial fruit set}\:(\%) \\&= [\text{Number of fruit set} / \text{Shoot length (cm)}]\times{100} \end{aligned}$$

Final fruit set percentage was computed after 60 days of full bloom with the same equation. Fruit drop % was determined using the Equation 4 as follow:

4 $$\begin{aligned} \text{Fruit drop}\:(\%)=\: &[\text{Initial fruit set percentage} \\&- \text{Final fruit set percentage} \\&/\: \text{Initial fruit set percentage}]\times{100} \end{aligned}$$

#### Yield

The harvest was conducted in the fourth week of September when around 75% of olive fruits had turned purple colour. The yield of each tree was weighed individually, and the total yield was recorded in tonne per acre.

#### Physical fruit characteristics

On harvest time (last week of September), 10 fruits from each replicate (30 per treatment) were picked at random from treated trees. The following fruit characteristics were evaluated: Fruit weight, seed weight, and flesh weight (g) were estimated by a digital balance. Fruit volume (cm³) was determined by a measuring cylinder. Flesh thickness, fruit diameter, and fruit length were measured with a digital caliper. Flesh/seed ratio was computed with the Equation 5:

5 $$\text{Flesh/seed ratio} = \text{[Pulp weight (g) / (Seed weight (g)]}$$

Fruit shape index was determined using the Equation 6:

6 $$\text{Fruit shape index} = \text{[Fruit length (cm) / Fruit diameter (cm)]}$$

#### Leaf and fruit mineral contents

During the last week of September in each ‘off-year’ season, 10 leaves were collected at random from the 5^th^ node of the selected shoots in each replicate, giving a total of 30 leaves per treatment. The samples were washed, dried, powdered, and digested according to Cottennie [33]. Total nitrogen was estimated using the micro-Kjeldahl method [34], potassium content was determined by a flame photometer [35], and phosphorus was measured according to Jackson [36]. Boron (B) was determined by the curcumin method by Dible et al. [37]. Calcium (Ca) was determined using an atomic absorption spectrophotometer [38]. At the same time, 10 fruits per tree (30 per treatment) were harvested at random from all sides of the trees. The fruit flesh was then processed in the same manner as described for the leaf samples.

#### Vegetative growth characteristics

Using a soft tape measure, shoot length was measured at two time points: December (prior to the experimental period) and the subsequent October (at the end of each ‘off-year’ season). These measurements were taken during the last week of October to assess vegetative growth parameters. From these data, the percentage increase in shoot length was determined. The number of new shoots per tagged shoot was counted, and their length was recorded. Leaves fresh and dry weight were measured using 10 leaves collected from each tree. All samples were taken from the 5^th^ node from the shoot apex, giving a total of 30 leaves per treatment. The fresh weight was measured utilizing a digital balance, whereas dry weight was determined after the samples were dried in the oven at 70 °C for 72 h. Leaf area was calculated from 30 mature leaves collected for each treatment, using the Equation 7 established by Ahmed and Morsy [39]:

7 $$\text{Leaf area}\:\left(\mathrm{cm}^{2}\right)=0.53\left(\text{Leaf length}\times\:\text{Leaf width}\right)+1.66$$

#### Moisture and oil content

The moisture and oil content of the ‘Picual’ olive fruits from all treatments were determined following the method outlined by the A.O.A.C [40]. For oil extraction, ‘Picual’ olive fruits from each treatment were harvested, crushed, and subsequently pressed using a Carver laboratory hydraulic press. Pressure was applied gradually, reaching 12.000 lb/in², and maintained for 30 min per sample. The resulting olive extract was collected and transferred to a separator funnel, where it was left until complete oil separation occurred. The oil layer was then collected and centrifuged. After centrifugation, the oil was dried and filtered through a cotton filter. Finally, it was stored in brown glass bottles at -20 °C until further analysis.

#### Quality parameters of olive oil

Free fatty acids (%), peroxide value (mEq O₂/kg oil), and saponification value (mg KOH/g oil) were determined according to AOAC [40]. Specific absorption coefficients (K_232_, K_270_, and ΔK) were measured following the methods described by the IOC [15]. Total phenol content was analysed using the method of Hrncirik and Fritsche [41], and results were expressed as mg of caffeic acid equivalent per kilogram of oil. Total pigment content, including chlorophylls and carotenoids, was determined colorimetrically. Measurements were performed using a UV-Visible spectrophotometer (JENWAY 6405 UV/Vis., England), following the method described by Mínguez Mosquera et al. [42]. Results were expressed as mg per kilogram of oil. The antioxidant activity of phenolic extracts obtained from all olive oil samples was evaluated using the stable 2.2-diphenyl-1-picrylhydrazyl (DPPH) radical scavenging assay, as described by Blois [43].

### Measurements during the ‘on-year’ season

The same trees that had received NPK and CaB nano-fertilizer treatments during the ‘off-year’ season were observed in the following ‘on-year’ season. The subsequent parameters were then recorded:

#### Yield

Yield evaluation for the ‘on-year’ season was carried out at the last week of October when approximately 75% of olive fruits had attained the purple colour. The total yield was recorded in tonne per acre.

#### Alternate bearing severity

To assess yield variation between successive seasons, the alternate bearing index was computed following the methodology of Monselise and Goldschmidt [44] using the Equation 8:

8 $$\begin{aligned} \text{Alternate bearing severity}\:(\%) =\:& [\text{Yield of the `on-year'} \\&- \text{Yield of the `off-year'} \\&/\: \text{Yield of the `on-year'}]\times{100}\end{aligned}$$

### Statistical analysis

This experiment was arranged in a randomized complete block design (RCBD). Each treatment consisted of three replicates, with one tree per replicate. Different experimental units were used in each season. Therefore, season was included as a fixed factor with two levels, and replicates were nested within seasons. Data were subjected to analysis of variance using R statistical program software version 4.5.2 [45]. The data were analysed using the following linear mixed model: $\mathrm{Y}_\mathrm{ijkl} = \mu + \mathrm{S}_\mathrm{i} + \mathrm{T}_\mathrm{j} + \left(\mathrm{S}\times\mathrm{T}\right)_\mathrm{ij} + \mathrm{R}_\mathrm{k}\left(\mathrm{S}_\mathrm{i}\right)+\in_\mathrm{ijkl}$, where $\mathrm{Y}_\mathrm{ijkl}$ is the response variable, µ is the fixed overall mean, $\mathrm{S}_\mathrm{i}$ is the fixed effect of season, $\mathrm{T}_\mathrm{j}$ is the fixed effect of treatment, $\left(\mathrm{S}\times\mathrm{T}\right)_\mathrm{ij}$ is the season × treatment interaction, $\mathrm{R}_\mathrm{k}\left(\mathrm{S}_\mathrm{i}\right)$ is the random effect of replicate nested within season, and $\in_\mathrm{ijkl}$ is the residual error. Since the interaction between season and treatment was not significant for most variables, the data were presented as combined means across seasons. However, for leaf N, leaf Ca, and yield of ‘off-year’ variables, where the interaction was significant, the results were presented as combined means in the main manuscript and as season-specific means in the supplementary files (Supplementary Data S3). Means were compared according to Duncan’s multiple range tests at significant level of 5% [46]. PCA was conducted to visualize the relationships between treatments and the studied variables [47]. A heatmap with hierarchical clustering was generated to provide an overview of the data matrix, grouping similar treatments and variables based on their expression patterns, following the method of Metsalu and Vilo [48].

## Results

### NPK-NF and CaB-NF characterization

#### Transmission electron microscopy (TEM)

**Fig. 1:**
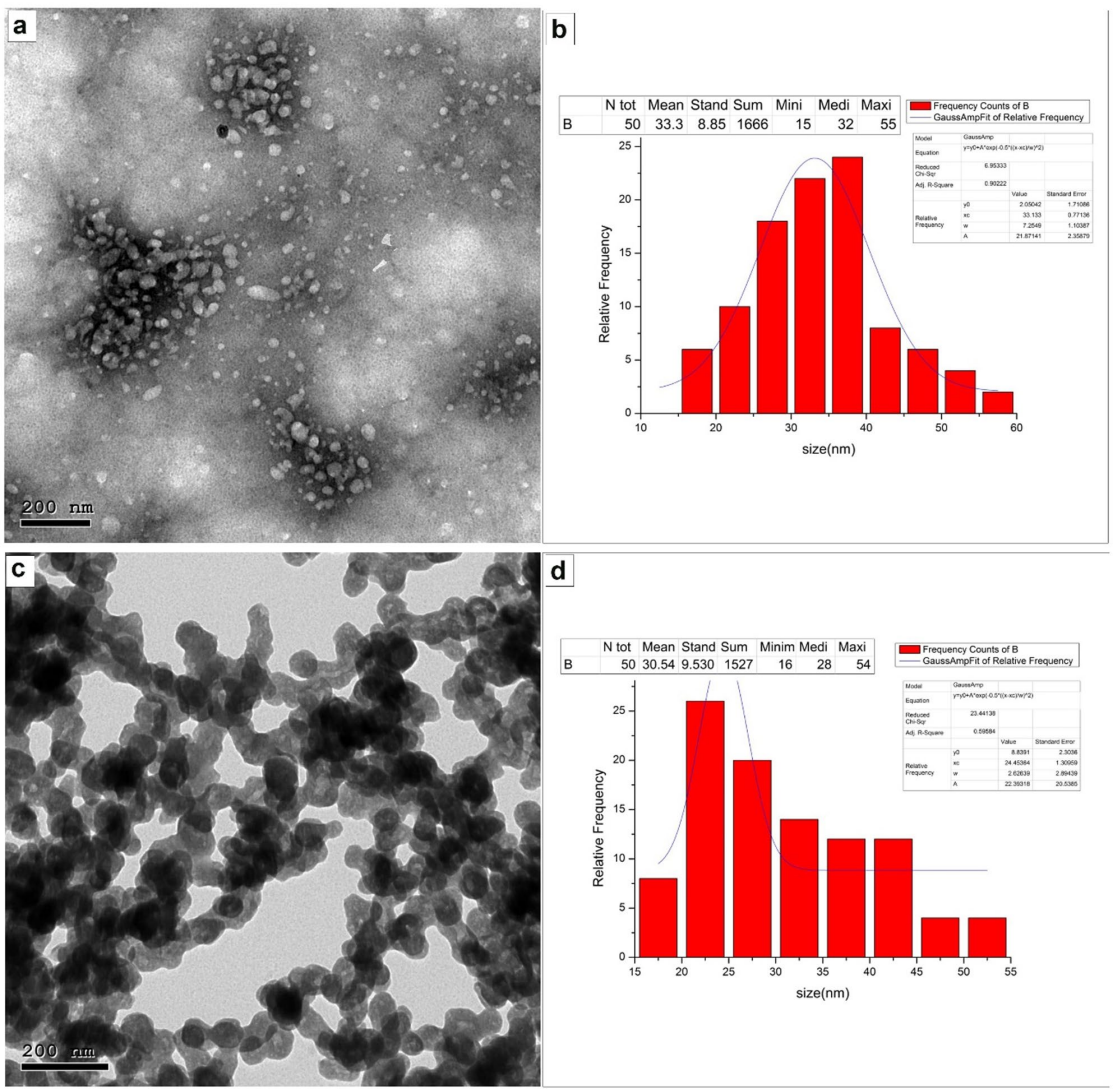
Morphological and particle size characterization of the synthesized nano-fertilizers. **a** Transmission electron microscopy (TEM) image of NPK nano-fertilizers (NPK-NF); **b** corresponding particle size distribution histogram of NPK-NF; **c** TEM image of calcium-boron nano-fertilizers (CaB-NF); and (**d**) corresponding particle size distribution histogram of CaB-NF used in the present study. Panels (**a**) and (**b**) are reproduced from our previous publication [22], which was published under the Creative Commons Attribution 4.0 International License (CC BY 4.0)

The NPK formulation underwent characterization via transmission electron microscopy (TEM), as documented in our earlier investigation [22]. Transmission electron microscopy (TEM) analysis provided insight into the structural morphology and size of the NPK nano-fertilizers. The nanoparticles exhibited a predominantly spherical or granular morphology with a moderate degree of polydispersity (Fig. 1a).

The corresponding size distribution histogram in Fig. 1b illustrates the frequency of the measured particle diameters. Analysis of 50 particles revealed a mean particle size of 33.3 nm, with a standard deviation of 8.85 nm. The particle sizes ranged from a minimum of 16 nm to a maximum of 52 nm, with the most frequent size (median) centered at 35 nm, confirming the nanoscale dimensions of the synthesized fertilizers. Parallel analytical procedures were likewise conducted for the CaB-nano-fertilizers (CaB-NF; Fig. 1c and d). Figure 1c displays a transmission electron microscopy (TEM) micrograph that reveals the structural attributes and morphology of the synthesized nanomaterials. The particles exhibit a range of shapes, from nearly spherical to slightly elongated forms, and appear well-dispersed with minimal aggregation.

Figure 1d quantifies these observations through a histogram of the particle size distribution. The analysis, based on a sample size of 50 particles, indicates a mean diameter of approximately 30.5 nm with a standard deviation of 9.5 nm, confirming the nanoscale dimensions of the synthesized fertilizers. The distribution spans from a minimum size of 16 nm to a maximum of 54 nm, providing a comprehensive view of the particle size range employed in this study.

#### Energy dispersive X-ray (EDX) spectroscopy

**Fig. 2:**
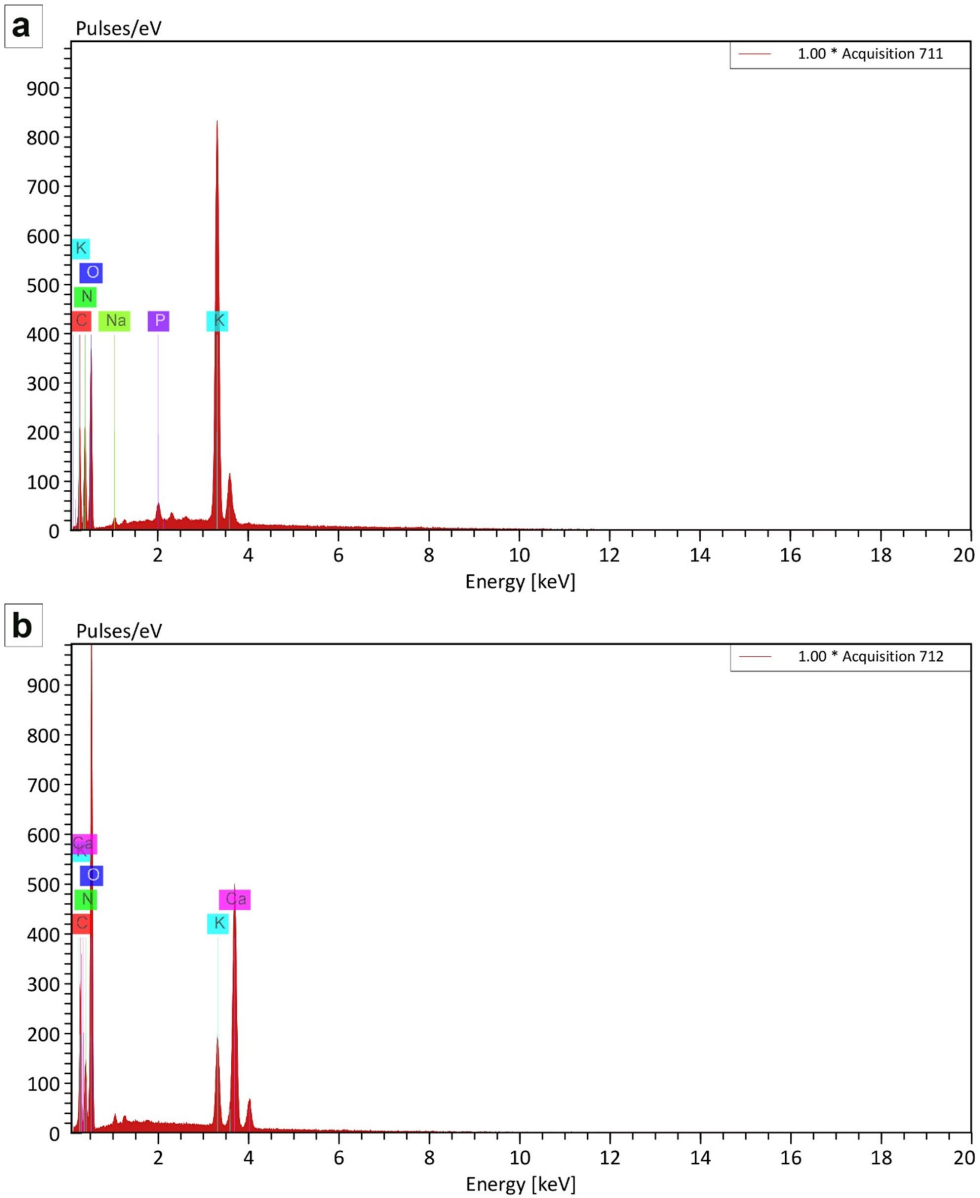
Representative EDX spectrum and elemental quantification of the synthesized (**a**) NPK nano-fertilizers (NPK-NF) and (**b**) calcium-boron nano-fertilizers (CaB-NF)

Energy Dispersive X-ray (EDX) spectroscopy provided quantitative verification of the elemental constitution within the synthesized NPK nano-fertilizer (Fig. 2a). The analysis confirmed the integration of all targeted macronutrients, namely nitrogen, phosphorus, and potassium.

Nitrogen emerged as the predominant element, registering an atomic concentration of 37.04 at% (31.10 wt%). Potassium was measured at 7.77 at% (18.20 wt%), while phosphorus appeared at a notably lower atomic percentage of 0.38 at% (0.70 wt%). Substantial contributions from carbon (11.70 at%) and oxygen (42.58 at%) were recorded, which points to the existence of an organic framework or a carbon-based matrix inherent to the formulation process. The detection of trace sodium (0.55 at%) is attributable to residual precursors from the synthetic pathway. The pronounced atomic fraction of nitrogen, coupled with the verified presence of potassium and phosphorus, substantiates the classification of the material as an NPK-grade nanofertilizer. The comparatively modest weight% of phosphorus implies its likely incorporation within a compound structure or encapsulation by the organic phase, consistent with the intended design for sustained nutrient release.

Also, EDX spectroscopy provided a quantitative assessment of the elemental profile of the synthesized calcium-boron (CaB) particulates (Fig. 2b). The analysis identified oxygen (78.55 wt%), nitrogen (18.42 wt%), calcium (14.69 wt%), carbon (12.97 wt%), and potassium (3.86 wt%) as the major constituents.

The elevated atomic proportion of oxygen (63.19 at%), combined with substantial carbon and nitrogen content, indicates the formation of organo-mineral complexes, likely arising from organic matrices or surfactants employed during synthesis. Successful detection of calcium (11.43 wt%, 4.72 at%) verifies its effective incorporation into the particulate architecture. Boron, however, remained below the instrument’s detection limit; this absence could stem from concentration levels beneath the threshold, incorporation into non-crystalline or organically bound states not amenable to EDX interrogation, or lattice substitution that yields no distinct spectral response.

#### Fourier-transform infrared (FT-IR) spectroscopy

**Fig. 3:**
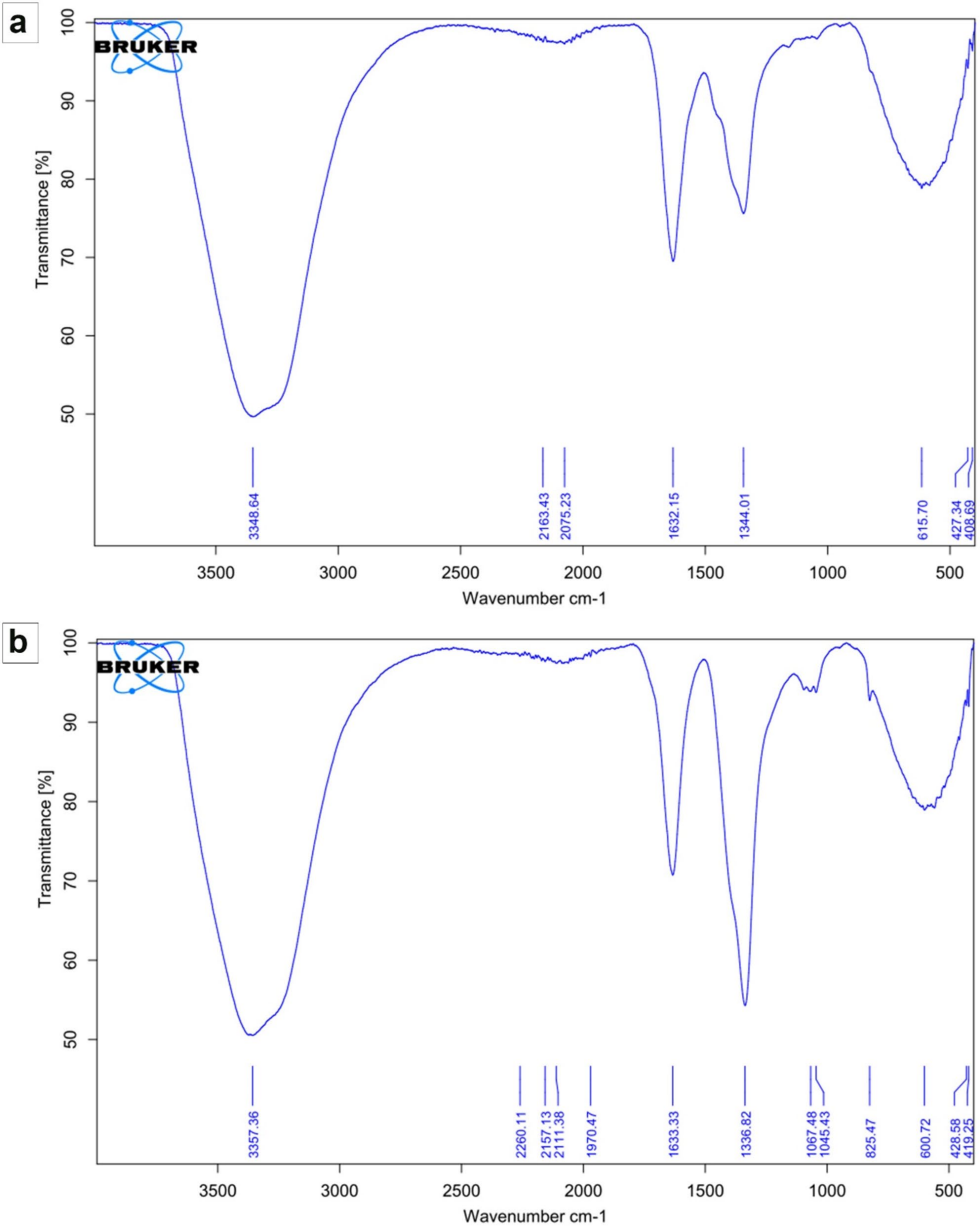
Representative FT-IR spectra of the synthesized (**a**) NPK nano-fertilizers (NPK-NF) and (**b**) calcium-boron nano-fertilizers (CaB-NF)

Fourier-transform infrared (FT-IR) spectroscopy was employed to elucidate the functional group architecture and bonding environment within the NPK-NF (Fig. 3a).

A prominent, broad absorption centered near 3349 cm⁻¹ arises from overlapping O–H and N–H stretching vibrations, indicative of hydroxyl groups, adsorbed moisture, and likely amine or amide functionalities. The presence of a well-defined band at 2163 cm⁻¹ is attributed to nitrile (–C ≡ N) or isocyanate (–N = C=O) stretching modes, pointing to the inclusion of specific nitrogenous organic species. A strong absorption at 1632 cm⁻¹ corresponds to carbonyl stretching (amide I) or asymmetric carboxylate vibrations, confirming the integration of carbonyl moieties into the structural framework. Supplementary bands at 1344 cm⁻¹ and 616 cm⁻¹ are assigned to C–N stretching and out-of-plane deformations, respectively, providing additional evidence for an organic matrix rich in nitrogen. Spectral features in the low-energy region, notably the absorptions at 428 cm⁻¹ and 409 cm⁻¹, are consistent with metal–oxygen (M–O) vibrational modes, supporting the coordination of potassium and possibly phosphorus within a mineral-organic hybrid structure. Collectively, the FT-IR data substantiate the composite character of the formulation, wherein organic components bearing amine, carbonyl, and nitrile groups are intimately associated with inorganic nutrient species. FT-IR spectroscopy also provided insight into the chemical bonding and structural characteristics of the CaB-NF (Fig. 3b), revealing a hybrid organic-inorganic composite architecture. A broad absorption centered around 3335 cm⁻¹ arises from overlapping O–H and N–H stretching vibrations, indicating the presence of hydroxyl groups, adsorbed water, and potential amine functionalities. Sharp bands observed at 2107 cm⁻¹ and 2111 cm⁻¹ correspond to nitrile (–C ≡ N) or isocyanate (–N = C=O) stretching modes, suggesting the involvement of nitrogenous organic species. A strong absorption at 1633 cm⁻¹ is attributed to carbonyl (C = O) stretching, consistent with amide I or carboxylate groups, while the accompanying band at 1537 cm⁻¹ reflects N–H bending characteristic of amide II vibrations. Absorptions within the 1000–1100 cm⁻¹ region, specifically at 1067 cm⁻¹ and 1045 cm⁻¹, are assigned to C–O stretching from alcohol or ether moieties, with potential contributions from B–O stretching in borate complexes. Notably, low-energy bands at 600 cm⁻¹, 423 cm⁻¹, and 419 cm⁻¹ correspond to metal–oxygen (M–O) lattice vibrations, confirming the successful incorporation of calcium into the inorganic framework. The absence of a pronounced band near 1350 cm⁻¹, typically associated with boron–oxygen stretching, implies that boron is either present in a non-crystalline state, complexed within the organic phase, or below the spectroscopic detection limit.

### Data of the ‘off-year’ season

#### Effect of NPK-NF and CaB-NF on flowering parameters of ‘Picual’ olive

**Fig. 4:**
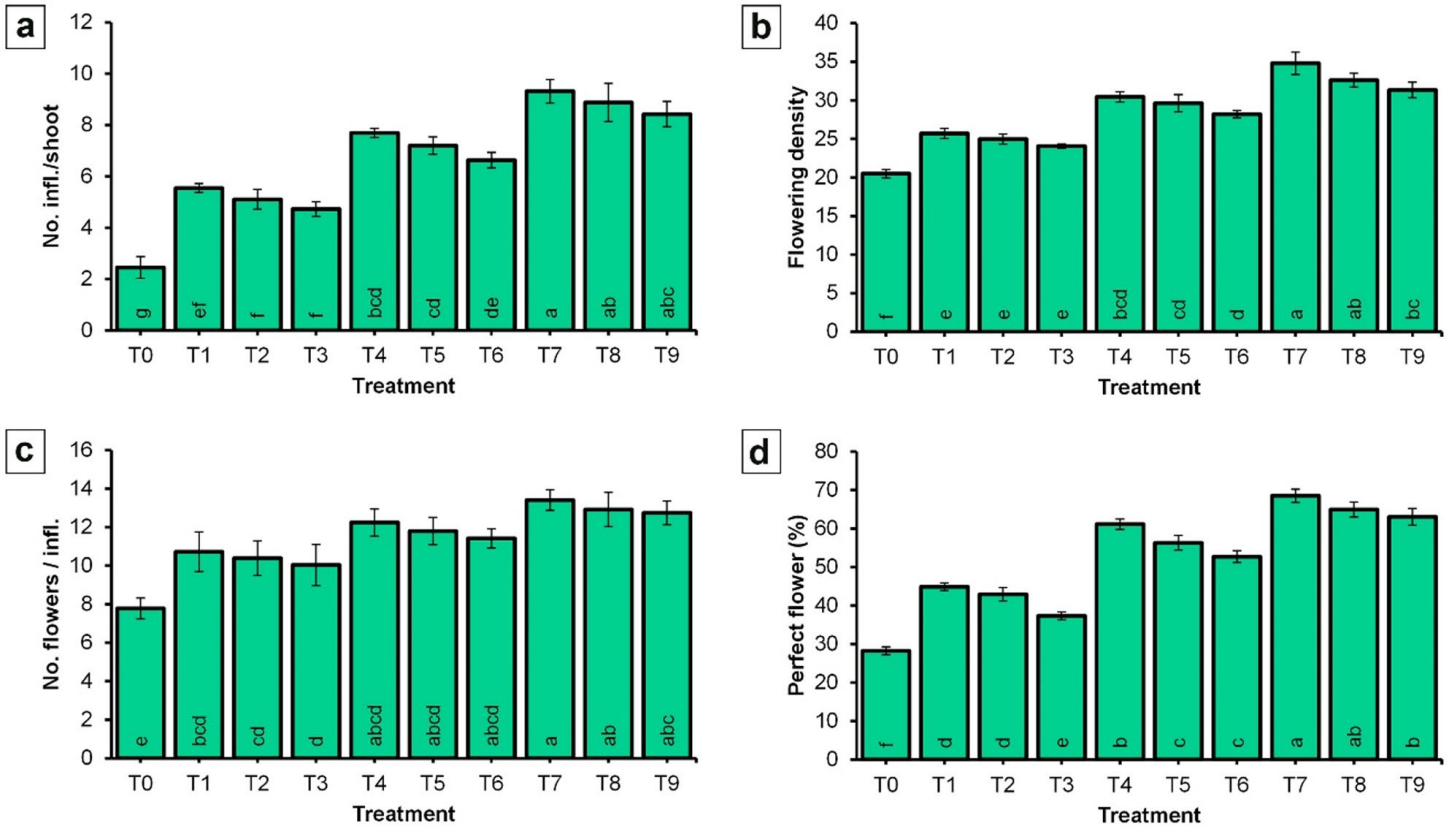
Effect of spraying NPK and CaB nano-fertilizers on flowering parameters of ‘Picual’ olive cultivar in the ‘off-year’ season. **a** Number of inflorescences per shoot; **b **Flowering density; **c** Number of flowers per inflorescence; (d) Perfect flower percentage. Values represent combined means across seasons, as the season × treatment interaction was not significant. Error bars denote standard error (SE). Within each parameter, different lowercase letters at the base of each histogram indicate significant differences according to Duncan’s multiple range test (*p* < 0.05)

The data presented in Fig. 4 demonstrate that the application of NPK-NF combined with CaB-NF significantly enhanced the flowering characteristics of ‘Picual’ olive trees compared to the control trees.

The most pronounced effect was observed with the treatment comprising NPK at 4 mL/L and CaB at 2 mL/L (T7), which resulted in the highest significant values across all measured parameters. Specifically, this treatment yielded 9.31 inflorescences per shoot, a flowering density of 34.81, 13.41 flowers per inflorescence, and a perfect flower percentage of 68.51%. In contrast, the control treatment (T0) recorded the lowest values for all flowering parameters, with 2.45 inflorescences per shoot, a flowering density of 20.49, 7.79 flowers per inflorescence, and a perfect flower percentage of 28.25%.

#### Effect of NPK-NF and CaB-NF on fruiting parameters and yield of ‘Picual’ olive

**Fig. 5:**
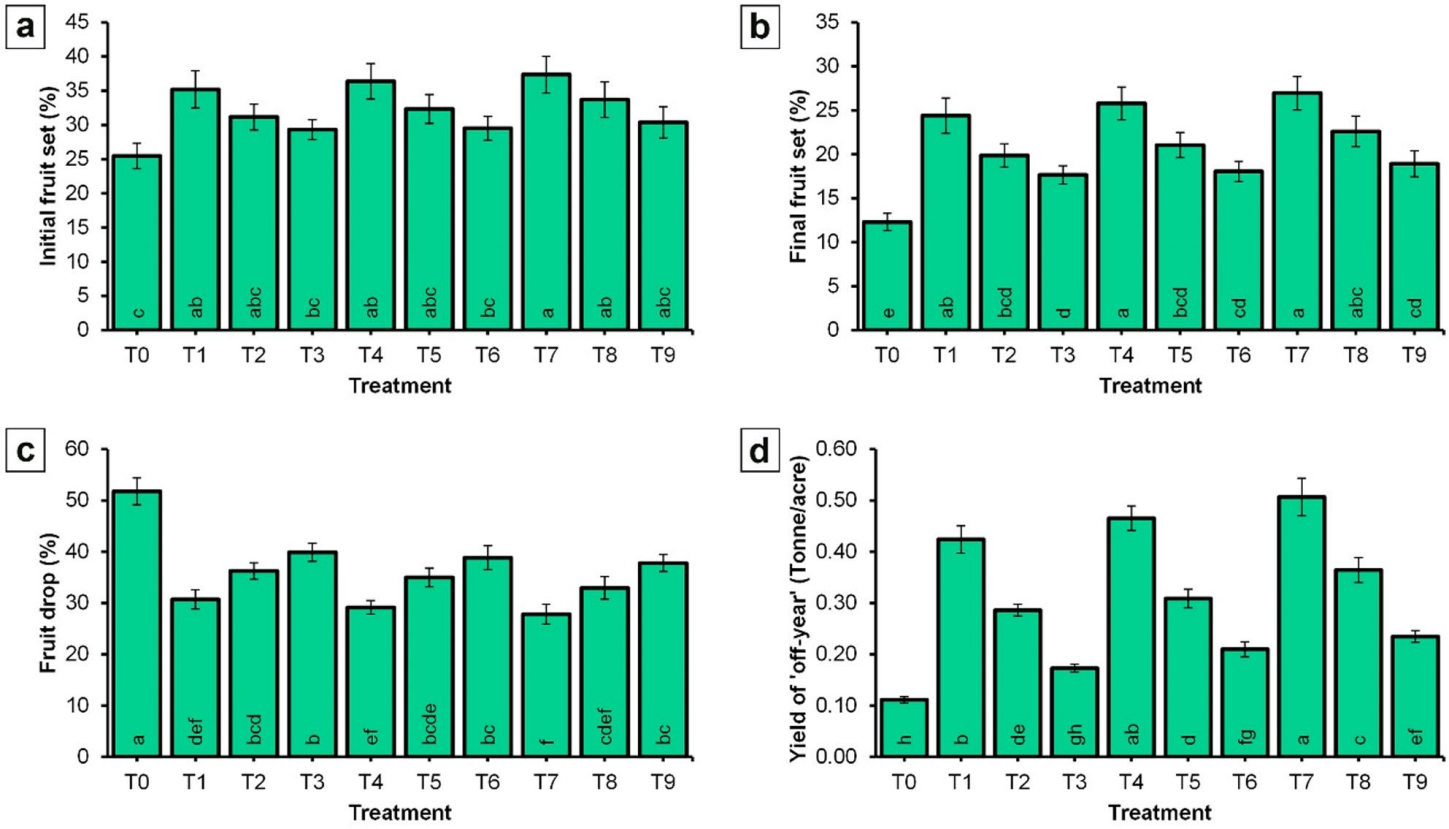
Effect of spraying NPK and CaB nano-fertilizers on fruiting parameters and yield of ‘Picual’ olive cultivar in the ‘off-year’ season. **a** Initial fruit set percentage; **b** Final fruit set percentage; **c** Fruit drop percentage; **d** Yield. Values represent combined means across seasons, as the season × treatment interaction was not significant. Error bars denote standard error (SE). Within each parameter, different lowercase letters at the base of each histogram indicate significant differences according to Duncan’s multiple range test (*p* < 0.05)

As is evident from the data in Fig. 5, initial fruit set (Fig. 5a), final fruit set (Fig. 5b), and fruit drop percentage (Fig. 5c) were all significantly influenced by the applied treatments. The highest initial fruit set (37.37%) and final fruit set (26.95%) were observed in trees sprayed with T7. In contrast, the control trees (T0) recorded the lowest initial fruit set (25.47%) and final fruit set (12.31%).

Regarding fruit drop (Fig. 5c), the control treatment exhibited the highest percentage (51.77%), whereas the treatment T7 (NPK-NF (4 mL/L) + CaB-NF (2 mL/L)) showed the lowest rate (27.83%). Yield was also markedly affected by the application of NPK-NF and CaB-NF (Fig. 5d). Moreover, T7 produced the highest yield, at 0.51 tonnes per acre, while the control treatment resulted in the lowest yield, at just 0.11 tonnes per acre.

#### Effect of NPK-NF and CaB-NF on physical fruit characteristics of ‘Picual’ olive

**Fig. 6:**
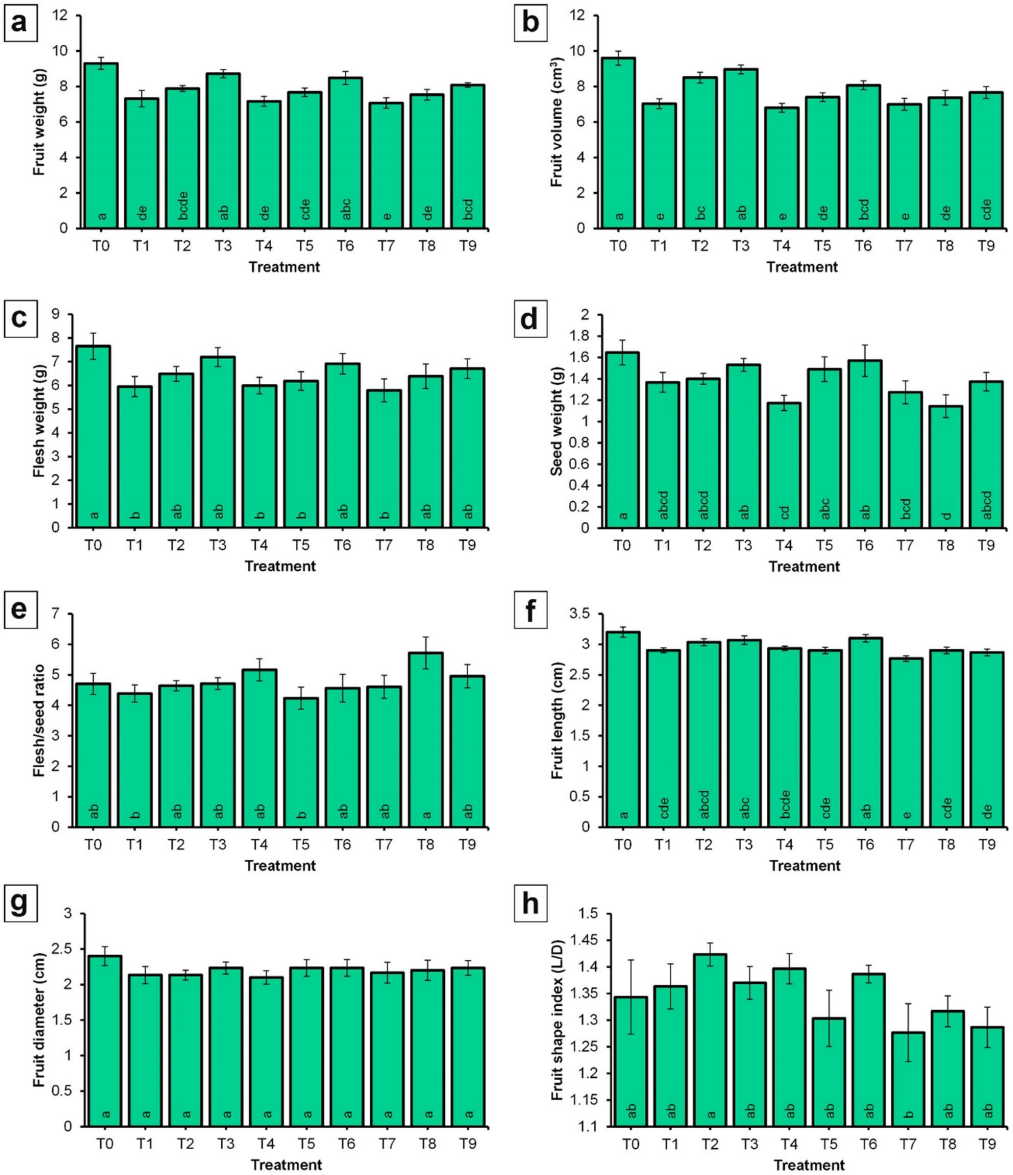
Effect of spraying NPK and CaB nano-fertilizers on physical fruit characteristics of ‘Picual’ olive cultivar in the ‘off-year’ season. **a** Fruit weight; **b** Fruit volume; **c** Flesh weight; **d** Seed weight; **e** Flesh/seed ratio; **f** Fruit length; **g** Fruit diameter; **h** Fruit shape index. Values represent combined means across seasons, as the season × treatment interaction was not significant. Error bars denote standard error SE. Within each parameter, different lowercase letters at the base of each histogram indicate significant differences according to Duncan’s multiple range test (*p* < 0.05)

The data presented in Fig. 6 indicate that the applied treatments significantly influenced fruit weight, fruit volume, flesh weight, seed weight, and flesh-to-seed ratio in ‘Picual’ olive trees. The unsprayed control trees recorded the highest values for fruit weight (9.30 g), fruit volume (9.33 cm³), flesh weight (7.65 g), and seed weight (1.65 g).

In contrast, the T7 treatment (NPK-NF (4 mL/L) + CaB-NF (2 mL/L)) produced the lowest values for fruit weight (7.07 g), fruit volume (7.00 cm³), and flesh weight (5.79 g). The lowest seed weight (1.14 g) was observed in trees treated with NPK-NF at 4 mL/L combined with CaB-NF at 3 mL/L (T8). Regarding the flesh-to-seed ratio, only slight differences were observed among treatments. The highest ratio (5.72) was also recorded with T8 treatment, whereas the lowest ratio (4.23) was obtained from the treatment with NPK-NF at 3 mL/L combined with CaB-NF at 3 mL/L (T5). Also, Fig. 6 presents the effects of the treatments on fruit dimensions. The control treatment (T0) yielded the highest significant values for both fruit length (3.20 cm) and fruit diameter (2.40 cm), followed by T6 with a value equal to 3.10 cm. Conversely, the lowest fruit length (2.77 cm) was recorded for the treatment with NPK-NF at 4 mL/L and CaB-NF at 2 mL/L (T7). In contrast to fruit length, the fruit diameter and fruit shape index did not vary significantly across treatments, as shown in Fig. 6g and h.

#### Effect of NPK-NF and CaB-NF on leaf and fruit mineral contents of ‘Picual’ olive

**Fig. 7:**
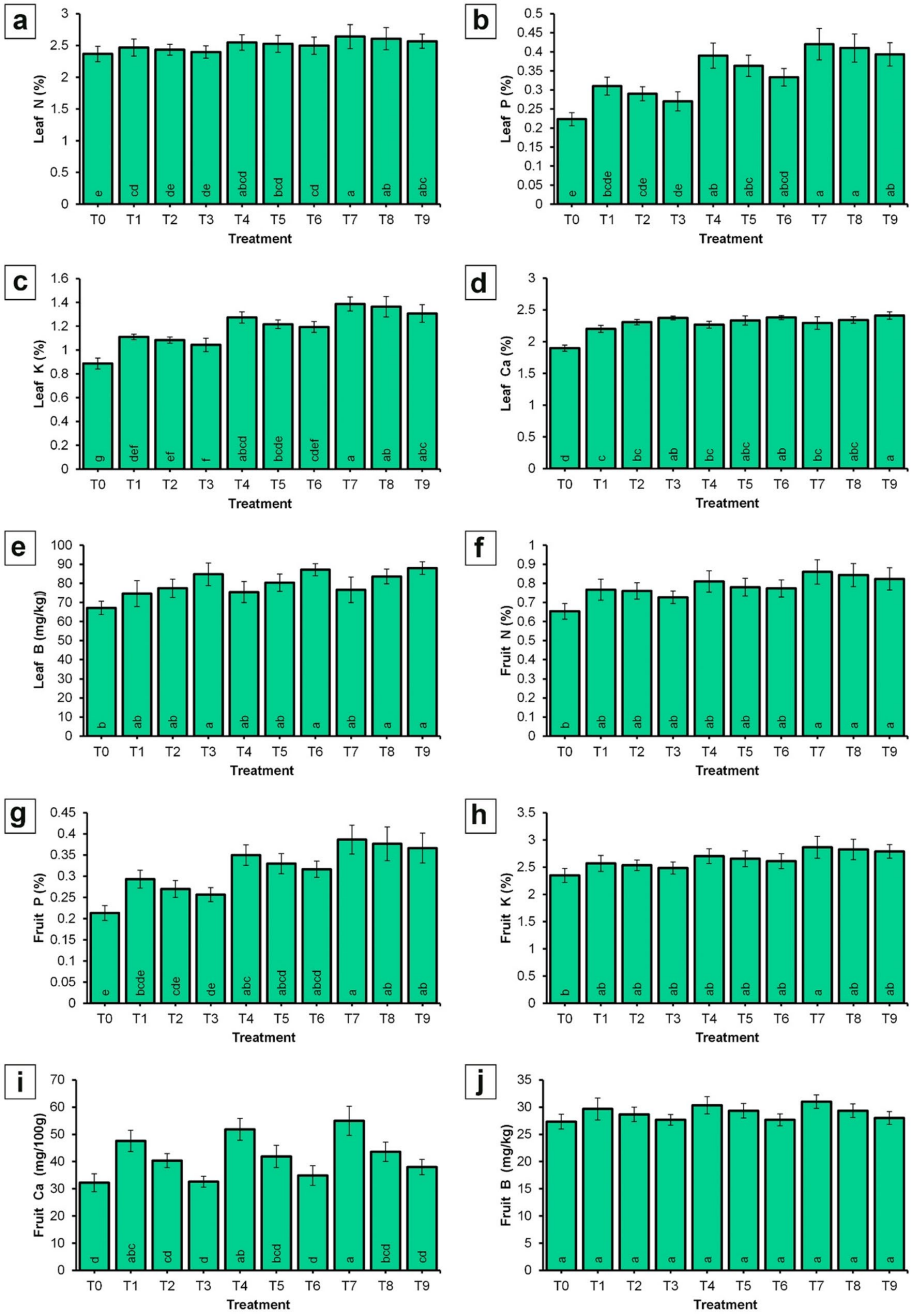
Effect of spraying NPK and CaB nano-fertilizers on N, P, K, Ca, and B content in leaves (Panels **a**‒**e**) and fruits (Panels **f**‒**j**) of ‘Picual’ olive cultivar during the ‘off-year’ season. Values represent combined means across seasons. The season × treatment interaction was not significant (NS) for most variables; however, a significant interaction was detected for leaf N and Ca%. For these variables, season-specific means are also provided in the supplementary material (Supplementary Data S3). Error bars denote standard error SE. Within each parameter, different lowercase letters at the base of each histogram indicate significant differences according to Duncan’s multiple range test (*p* < 0.05).

The data presented in Fig. 7 illustrate that the application of NPK-NF combined with CaB-NF significantly increased the leaf content of nitrogen, phosphorus, potassium, calcium, and boron compared to the control. Among the treatments evaluated, T7 (NPK-NF (4 mL/L) + CaB-NF (2 mL/L)) resulted in the highest significant concentrations of leaf nitrogen (2.64%), phosphorus (0.42%), and potassium (1.39%). In contrast, T9 comprising NPK-NF at 4 mL/L plus CaB-NF at 4 mL/L produced the highest leaf calcium (2.41%) and boron (88.00 mg/kg) levels. Conversely, the control treatment recorded the lowest values for all nutrients measured: nitrogen (2.37%), phosphorus (0.22%), potassium (0.89%), calcium (1.9%), and boron (67.11 mg/kg).

It is evident from the data in Fig. 7 that the NPK-NF and CaB-NF treatments also had a beneficial effect on the nutrient composition of the fruit flesh. The highest concentrations of nitrogen (0.86%), phosphorus (0.39%), potassium (2.87%), and calcium (54.99 mg/100 g) in the flesh were observed in response to spraying trees with NPK-NF at 4 mL/L combined with CaB-NF at 2 mL/L (T7).

In contrast, the control treatment recorded the lowest values for these nutrients, with nitrogen, phosphorus, potassium, and calcium concentrations of 0.65%, 0.21%, 2.35%, and 32.22 mg/100 g, respectively. Regarding boron content in the fruit flesh, the differences among all treatments were found to be statistically insignificant.

#### Effect of NPK-NF and CaB-NF on vegetative characteristics of ‘Picual’ olive

**Fig. 8:**
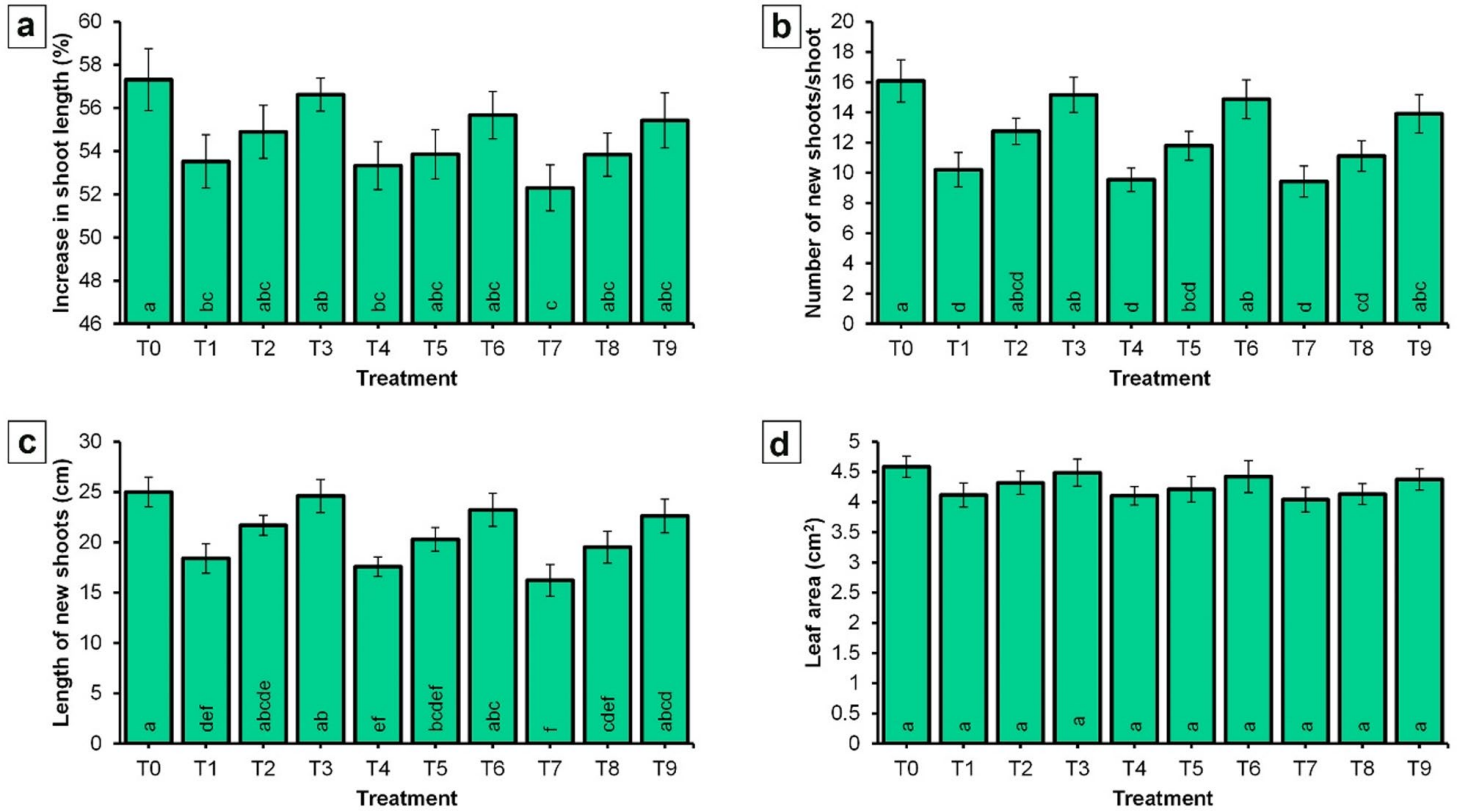
Effect of spraying NPK and CaB nano-fertilizers on vegetative growth parameters of ‘Picual’ olive cultivar in the ‘off-year’ season. **a** Increase in shoot length; **b** Number of new shoots per shoot; **c** Length of new shoots; **d** Leaf area. Values represent combined means across seasons, as the season × treatment interaction was not significant. Error bars denote standard error (SE). Within each parameter, different lowercase letters at the base of each histogram indicate significant differences according to Duncan’s multiple range test (*p* < 0.05)

The findings presented in Fig. 8 clearly indicate that untreated trees exhibited the greatest increase in shoot length, with a percentage increase of 57.32%. In contrast, the application of NPK-NF at 4 mL/L combined with CaB-NF at 2 mL/L (T7) resulted in the lowest shoot length increase, measuring 52.30%.

Furthermore, data demonstrates that control treatment (T0) produced the highest number of new shoots per shoot, with a mean of 16.08. Conversely, the lowest number of new shoots per shoot (9.42) was recorded for trees treated with NPK-NF at 4 mL/L plus CaB-NF at 2 mL/L (T7). Regarding the length of new shoots, the control treatment again yielded the highest significant value, at 25.00 cm, whereas the lowest significant value (16.22 cm) was also observed in T7 treatment. In contrast to the parameters described above, no significant differences were detected among treatments with respect to leaf area. The values for leaf area ranged from 4.04 to 4.58 cm² across all treatments.

#### Quality of olive oil

Foliar application of a combined NPK-NF and CaB-NF significantly increased the oil content of olives on a dry weight basis as indicated in Table 1. The most effective treatment, a 4:2 ratio of NPK-NF and CaB-NF (T7), yielded an oil content of 39.53%, compared to 33.88% in the unfertilized control (T0). This increase in oil accumulation was accompanied by a decrease in fruit moisture content, which declined to 65.54% by the nano-fertilization in T7 versus 68.43% in the control (T0), reflecting the typical inverse relationship between these parameters during ripening.

**Table 1:** Effect of spraying NPK and CaB nano-fertilizers on moisture and oil % of ‘Picual’ olive cultivar in the ‘off-year’ season

Treatment	Moisture content %	Oil content % (dry weight)
T0	68.43 ± 0.12^b^	33.88 ± 0.24^g^
T1	67.48 ± 0.11^d^	38.27 ± 0.08^c^
T2	66.73 ± 0.13^f^	35.34 ± 0.17^d^
T3	68.45 ± 0.12^b^	34.45 ± 0.06^f^
T4	68.02 ± 0.10^c^	38.85 ± 0.16^b^
T5	68.80 ± 0.10^a^	35.34 ± 0.08^e^
T6	67.38 ± 0.09^d^	35.90 ± 0.08^d^
T7	65.54 ± 0.08^g^	39.53 ± 0.03^a^
T8	67.04 ± 0.17^e^	38.01 ± 0.08^c^
T9	67.96 ± 0.22^c^	38.37 ± 0.06^c^

Results are reported as mean ± standard error (SE). Within each column, means denoted by the same lowercase superscript letter are not significantly different, as determined by Duncan’s Multiple Range Test at a significance level of *p* < 0.05

Table 2 revealed that all olive oil samples met the International Olive Council (IOC, 2022) standards for extra virgin classification. Free fatty acid (FFA) values remained well below the 0.8% threshold, ranging from 0.17% to 0.26%. Peroxide values (PV) were also within the acceptable limit (≤ 20 mEq O₂/kg), varying between 3.62 and 4.63 mEq O₂/kg oil. Similarly, the ultraviolet absorption coefficients (K232 and K270) complied with IOC standards, with values ranging from 0.750 to 0.880 and 0.040 to 0.053, respectively. Saponification values showed minimal variation (192.30–192.74 mg KOH/g oil), suggesting that the nano-fertilizer treatments had little to no effect on the average chain length of fatty acids.

**Table 2:** Quality characteristics of ‘Picual’ olive oil in response to NPK and CaB nano-fertilizers during the ‘off-year’ season

Treatment	*FFAs	*PV	*K_232_	K_270_	Δk	*SV
T0	0.24 ± 0.01^ab^	4.42 ± 0.01^bc^	0.85 ± 0.00^ab^	0.050 ± 0.00^a^	-0.0028 ± 0.00^a^	192.74 ± 0.07^a^
T1	0.17 ± 0.00^d^	4.63 ± 0.03^a^	0.88 ± 0.00^a^	0.051 ± 0.00^a^	-0.0014 ± 0.00^a^	192.61 ± 0.07^b^
T2	0.20 ± 0.00^cd^	3.96 ± 0.06^d^	0.79 ± 0.00^cde^	0.041 ± 0.00^b^	-0.0029 ± 0.00^a^	192.59 ± 0.07^b^
T3	0.21 ± 0.01^bc^	3.87 ± 0.17^de^	0.78 ± 0.00^de^	0.044 ± 0.00^b^	-0.0022 ± 0.00^a^	192.61 ± 0.03^b^
T4	0.25 ± 0.01^a^	4.30 ± 0.06^c^	0.84 ± 0.02^abc^	0.048 ± 0.00^a^	-0.0013 ± 0.00^a^	192.53 ± 0.05^bc^
T5	0.21 ± 0.01^bc^	4.26 ± 0.05^c^	0.78 ± 0.01^de^	0.047 ± 0.00^a^	-0.0030 ± 0.00^a^	192.56 ± 0.06^bc^
T6	0.24 ± 0.00^ab^	4.63 ± 0.04^a^	0.86 ± 0.00^a^	0.054 ± 0.00^a^	-0.0039 ± 0.00^a^	192.58 ± 0.06^bc^
T7	0.24 ± 0.01^ab^	3.62 ± 0.08^f^	0.75 ± 0.03^e^	0.043 ± 0.00^b^	-0.0055 ± 0.00^b^	192.46 ± 0.04^c^
T8	0.18 ± 0.00^cd^	4.54 ± 0.02^ab^	0.81 ± 0.00^bcd^	0.051 ± 0.00^a^	-0.0066 ± 0.00^c^	192.46 ± 0.02^c^
T9	0.26 ± 0.00^a^	3.74 ± 0.09^ef^	0.77 ± 0.00^de^	0.040 ± 0.00^b^	-0.0060 ± 0.00^c^	192.30 ± 0.09^d^

Within each column, means denoted by the same lowercase superscript letter are not significantly different, as determined by Duncan’s Multiple Range Test at a significance level of *p* < 0.05

*FFAs  Free fatty acids % as oleic acid, *PV*  Peroxide value mEq O₂/kg oil, *SV*  Saponification values mg KOH/g oil. Results are reported as mean ± standard error (SE)

In contrast to the oil content, the total phenolic content, measured as caffeic acid, was highest in the control sample (389.63 mg/kg) and decreased with increasing NPK-NF and CaB-NF application (Table 3). The lowest phenolic content (293.91 mg/kg) was observed in the treatment with the highest concentration of nano-nutrients (T9). This reduction was directly correlated with a decline in the antioxidant activity of the phenolic extract, which fell from 94.37% in control (T0) to 66.03% in T7 treatment. Conversely, pigment concentrations exhibited a positive response to nano-fertilization. Carotenoid and chlorophyll levels increased significantly, rising from 1.22 to 2.00 mg/kg in the control to 2.24 and 3.05 mg/kg, respectively, in T9 treatment.

**Table 3:** Pigments content, total poly phenols and antioxidant activity of ‘Picual’ olive oil in response to NPK and CaB nano-fertilizers during the ‘off-year’ season

Treatment	Total poly phenols (mg/kg)	Chlorophyll (mg/kg)	Chlorophyll (mg/kg)	Carotenoids (mg/kg)	Antioxidant activity of phenols extract (%)
T0	389.63 ± 0.87^a^	2.00 ± 0.01^i^	2.00 ± 0.01^i^	1.22 ± 0.02^f^	94.37 ± 0.43^a^
T1	337.71 ± 0.87^c^	2.39 ± 0.02^h^	2.39 ± 0.02^h^	1.52 ± 0.02^e^	87.25 ± 0.35^d^
T2	360.68 ± 0.87^b^	2.50 ± 0.00^g^	2.50 ± 0.00^g^	1.56 ± 0.04^e^	91.18 ± 0.77^b^
T3	359.09 ± 0.87^b^	2.59 ± 0.01^f^	2.59 ± 0.01^f^	1.61 ± 0.04^de^	89.47 ± 0.46^c^
T4	325.38 ± 0.87^d^	2.68 ± 0.01^e^	2.68 ± 0.01^e^	1.69 ± 0.04^d^	87.79 ± 0.43^d^
T5	316.50 ± 0.87^d^	2.80 ± 0.01^cd^	2.80 ± 0.01^cd^	1.82 ± 0.03^c^	79.83 ± 0.18^e^
T6	326.23 ± 0.87^cd^	2.84 ± 0.00^bc^	2.84 ± 0.00^bc^	1.89 ± 0.01^bc^	78.27 ± 0.52^f^
T7	267.92 ± 0.58^g^	2.75 ± 0.01^de^	2.75 ± 0.01^de^	1.88 ± 0.01^bc^	66.03 ± 0.45^i^
T8	280.48 ± 0.87^f^	2.89 ± 0.00^b^	2.89 ± 0.00^b^	1.96 ± 0.05^b^	71.45 ± 0.50^g^
T9	293.91 ± 0.87^e^	3.05 ± 0.00^a^	3.05 ± 0.00^a^	2.24 ± 0.04^a^	70.04 ± 0.35^h^

Results are reported as mean ± standard error (SE). Within each column, means denoted by the same lowercase superscript letter are not significantly different, as determined by Duncan’s Multiple Range Test at a significance level of *p* < 0.05

The fatty acid profile was also positively modulated by nano-fertilizer treatments (Table 4). The proportion of monounsaturated fatty acids, particularly oleic acid (C18:1), increased significantly from 63.94% in the control to 67.85% in T9 treatment. This was accompanied by a general decrease in polyunsaturated linoleic acid (C18:2), which ranged from 8.59% to 10.59% in treated samples compared to 10.52% in control.

**Table 4:** Relative percentage of fatty acids of ‘Picual’ olive oil in response to NPK and CaB nano-fertilizers during the ‘off-year’ season

Fatty acids	Treatment	T0	T1	T2	T3	T4	T5	T6	T7	T8	T9
Myristic acid (C14:0)		0.03	0.03	0.03	0.03	0.03	0.02	0.02	0.03	0.03	0.03
Palmitic acid (C16:0)		18.05	17.81	17.79	17.78	17.63	17.44	17.27	17.41	17.61	17.22
Palmitoleic acid (C16:1)		2.51	2.36	2.37	2.5	2.45	2.57	2.65	2.33	2.17	2.13
Maragic acid (C17:0)		0.06	0.06	0.06	0.05	0.09	0.04	0.04	0.08	0.04	0.06
Margoleic acid (C17:1)		0.11	0.12	0.12	0.16	0.12	0.1	0.1	0.15	0.1	0.13
Stearic acid (C18:0)		2.89	2.92	2.9	2.3	2.42	2.35	2.27	2.5	2.12	2.12
Oleic acid (C18:1)		63.94	64.22	64.63	65.69	65.67	66.1	67.38	65.85	67.56	67.85
Linoleic acid (C18:2)		10.52	10.59	10.18	9.75	9.81	9.61	8.6	9.84	8.64	8.59
Linolenic acid (C18:3)		1.1	1.06	1.05	1	1.01	1.06	1.04	1.01	1.02	1.03
Arachidic acid (C20:0)		0.44	0.47	0.48	0.4	0.39	0.38	0.33	0.4	0.35	0.43
Gadoleic acid (C20:1)		0.25	0.25	0.28	0.21	0.27	0.22	0.2	0.27	0.25	0.29
Behenic acid (C22:0)		0.1	0.11	0.11	0.13	0.11	0.11	0.1	0.13	0.13	0.12
C18:1/C18:2		6.07	6.06	6.35	6.74	6.69	6.88	7.83	6.69	7.82	7.89
Saturated fatty acids		21.57	21.4	21.37	20.69	20.67	20.34	20.03	20.55	20.28	19.98
Unsaturated fatty acids		78.43	78.6	78.63	79.31	79.33	79.66	79.97	79.45	79.74	80.02
Monounsaturated fatty acids		66.81	66.95	67.4	68.56	68.51	68.99	70.33	68.6	70.08	70.4
Poly unsaturated fatty acids		11.62	11.65	11.23	10.75	10.82	10.67	9.64	10.85	9.66	9.62
Iodine value (g *I*_2_/100 g oil)		82.20	82.32	81.96	82.07	82.18	82.43	81.76	82.28	81.50	81.69

Consequently, the oleic-to-linoleic acid ratio improved with fertilization. Levels of linolenic acid (C18:3) were near or slightly above the IOC maximum limit of 1.0% in some treatments, including the control, with values ranging from 1.00% to 1.10%. All other fatty acid values remained well within the established IOC standards.

### Data of the ‘on-year’ season

**Fig. 9:**
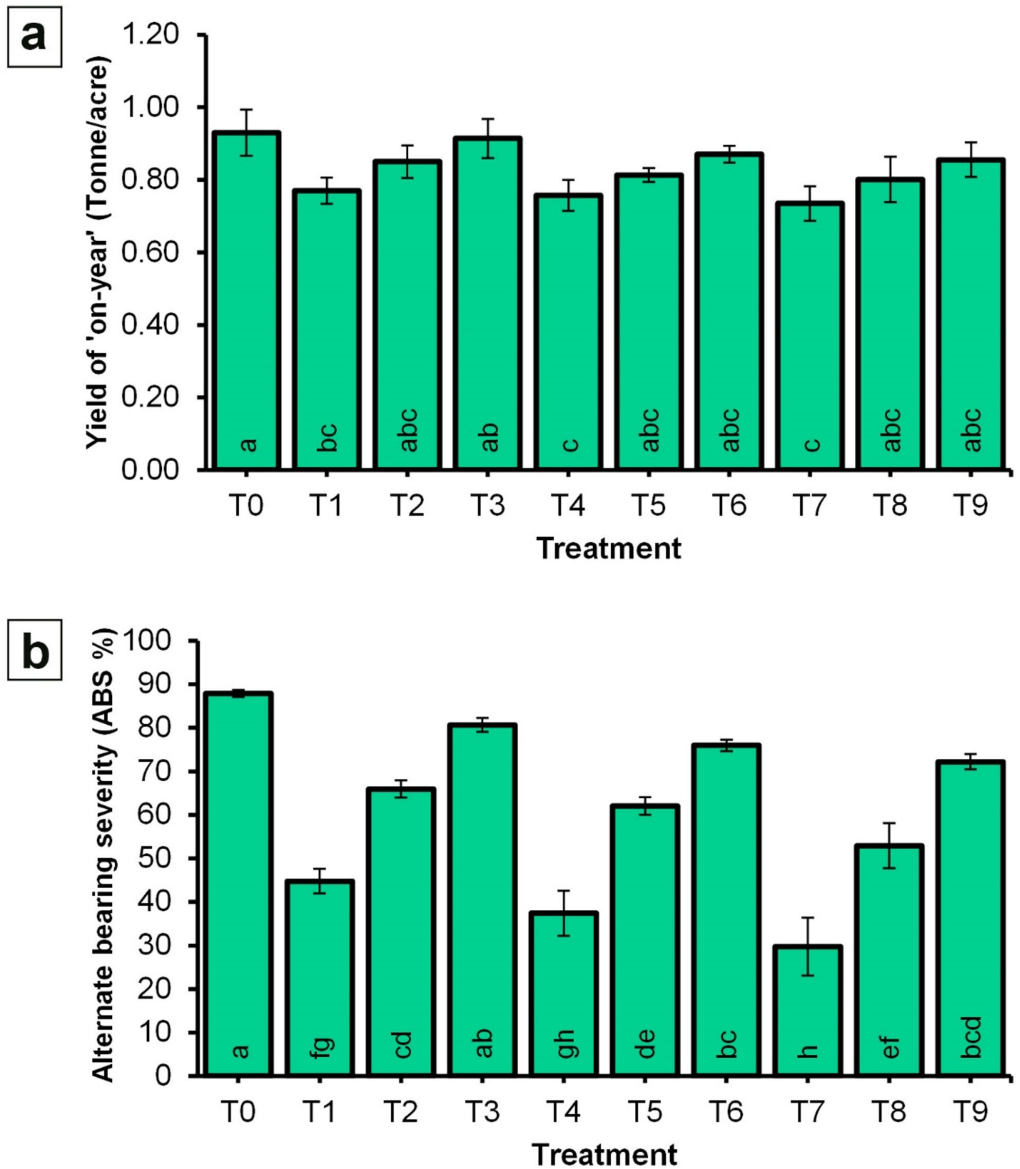
Effect of spraying NPK and CaB nano-fertilizers on yield and alternate bearing severity of ‘Picual’ olive cultivar in the ‘on-year’ season. **a** Yield; **b** Alternate bearing severity. Values represent combined means across seasons, as the season × treatment interaction was not significant. Error bars denote standard error (SE). Within each parameter, different lowercase letters at the base of each histogram indicate significant differences according to Duncan’s multiple range test (*p* < 0.05)

Yield during the ‘on-year’ season did not differ significantly among any of the treatments evaluated (Fig. 9). Values ranged from 0.74 to 0.93 tonnes per acre across the ten treatments. In contrast, alternate bearing severity was significantly influenced by the application of NPK-NF and CaB-NF treatments. The highest alternate bearing severity index was recorded for control treatment, with a value of 87. 86%. This was statistically comparable only to T3 (80. 67%) and T6 (75.95%). The lowest alternate bearing severity was observed in T7, which received NPK-NF at 4 mL/L combined with CaB-NF at 2 mL/L, yielding a severity index of 29.75%. This value was statistically similar to T4 (37.41%). Intermediate values were obtained for the remaining treatments, ranging from 44.78 to 72.22%.

### Multivariate analysis of treatment effects: principal component analysis and heatmap visualization

**Fig. 10:**
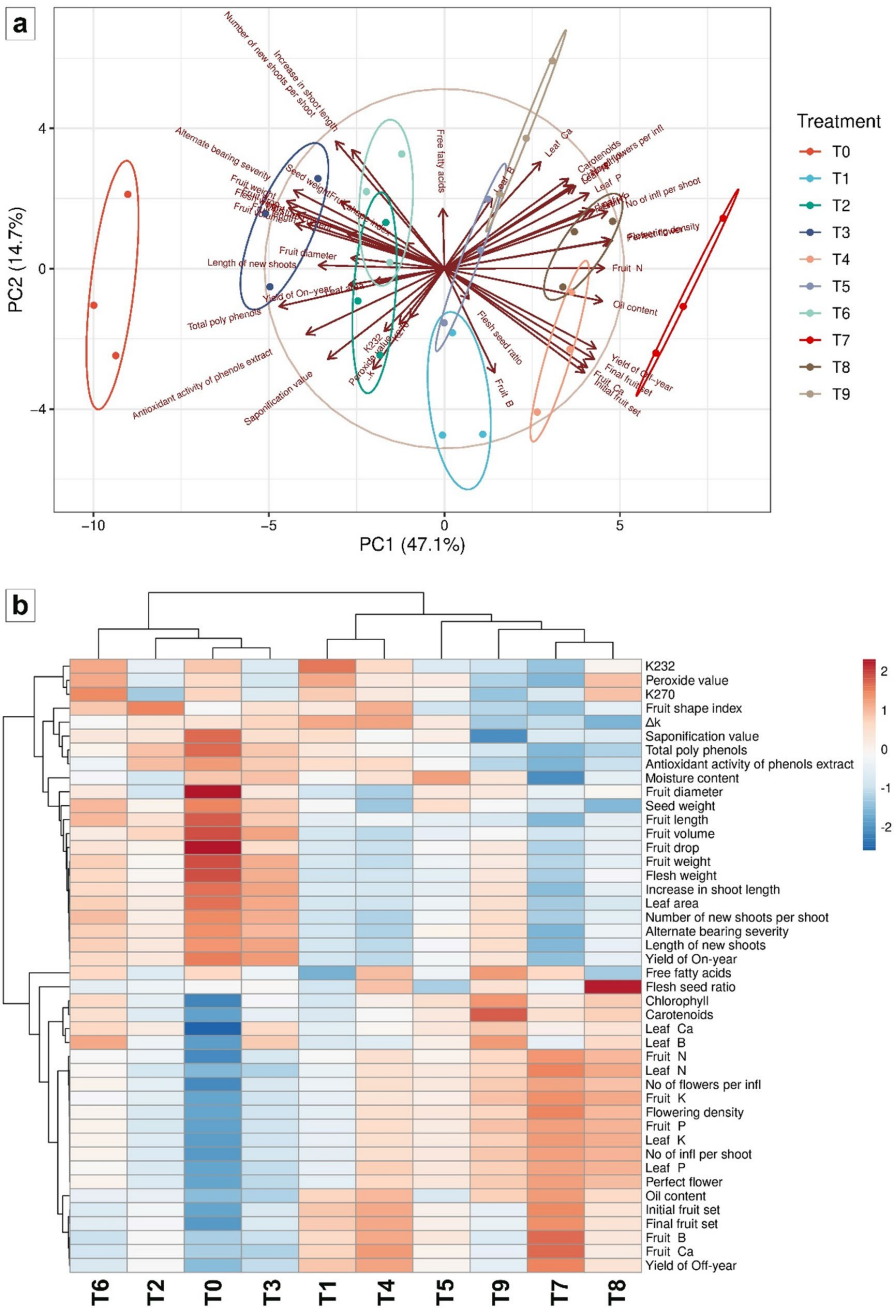
Principal component analysis (**a**) and heatmap visualization (**b**) of ten treatments (T0 to T9) based on 44 measured variables

Figure 10 presents the results of a principal component analysis (PCA) and a heatmap visualization based on 44 measured variables across the ten different treatments.

In the PCA score plot (Fig. 10a), the first two principal components explain 47.1% and 14.7% of the total variance, respectively, cumulatively accounting for 61.8% of the variation among treatments. The distribution of treatments along PC1 reveals a clear separation primarily driven by treatment intensity or gradient.

The heatmap in Fig. 10b provides a comprehensive overview of the normalized expression or intensity values for the 44 variables across the ten treatments. In this visualization, each column corresponds to a treatment and each row to an individual variable. The color scale, ranging from blue to red, represents the relative abundance or magnitude of each variable, with red indicating higher values and blue indicating lower values. Hierarchical clustering was applied to both treatments and variables, organizing the data into distinct groups that reflect underlying patterns. The clustering of treatments confirms the separation observed in the PCA plot. Both the PCA and heatmap reveal a clear differentiation between the control (T0) and the other treatments, particularly T7 (NPK-NF (4 mL/L) + CaB-NF (2 mL/L)). The clustering of variables identifies specific groups that exhibit coordinated responses across the treatment gradient, suggesting potential functional relationships or shared regulatory mechanisms. Together, these visualizations illustrate a structured response of the measured variables to the treatment gradient, with increasing treatment levels associated with distinct and progressively more pronounced changes in the multivariate profile.

## Discussion

Fertilizers formulated at the nanoscale are gaining attention as a novel strategy in crop nutrition. These products deliver both macronutrients like nitrogen, phosphorus, and potassium as well as essential micronutrients using particles or capsules measured in nanometers [49]. Because of their minute size, these materials possess a larger surface area relative to their volume and exhibit higher chemical reactivity. These characteristics improve their ability to dissolve, allow for a more gradual and controlled release of nutrients, and facilitate greater absorption by plant roots or leaves. Traditional fertilizers are prone to significant losses, whether through leaching deep into the soil, escaping as gases, or becoming chemically bound and unavailable to plants. Nano-fertilizers, by contrast, are engineered to release nutrients in closer alignment with the actual needs of the crop. This synchronization boosts the efficiency with which plants utilize applied nutrients and can translate into higher yields. Their design also offers the potential for more precise delivery, which could lessen the amount of fertilizer that runs off fields or contaminates water sources, tackling some of the persistent environmental problems linked to modern farming [50].

This study evaluated the application of NPK and calcium boron nano fertilizers (NPK-NF and CaB-NF) during the ‘off-year’ season as a strategy to improve flowering, fruit set, yield, and oil quality in the ‘Picual’ olive cultivar. The foundational role of nitrogen in olive reproductive physiology has been well documented in the literature. Chatzissavvidis et al. [51] provided early evidence linking nitrogen deficiency to a reduction in the number of flowers per inflorescence. Expanding on this, Therios [10] established that nitrogen is critical for promoting the formation of hermaphrodite flowers, noting that leaf nitrogen concentrations below 1% favor the development of staminate flowers, thereby diminishing fruit set potential. Subsequently, the availability of multiple macronutrients has been shown to regulate flowering intensity. Erel et al. [7] reported that the availability of nitrogen, phosphorus, and potassium directly influences the number of inflorescences produced per shoot. This was further refined by Erel et al. [52], who demonstrated that the availability of both nitrogen and phosphorus specifically affects the overall flowering intensity in olive trees. In addition to macronutrients, the role of micronutrients, particularly boron and calcium, is also pivotal. Perica et al. [14] demonstrated that foliar boron application can significantly increase the percentage of perfect flowers and improve subsequent fruit set in olive. This has been supported by more recent work with nano formulations. Vishekaii et al. [53] illustrated that foliar application of nano boron increased the number of perfect flowers in olive trees. Similarly, calcium plays a crucial role, as confirmed by El Hady et al. [54], who found that a late December spray of 0.5% chelated calcium significantly enhanced inflorescence production, flower number per inflorescence, and overall sex expression in olive. The flowering enhancements observed in the current study are likely attributable to the superior properties of the nano fertilizer formulations. Their primary effect is the enhancement of nutrient availability and uptake efficiency [50, 55–57]. Once absorbed, these nutrients underpin a range of physiological processes essential for reproductive development. Due to their high penetration efficiency, nano fertilizers can readily cross cellular membranes to reach functional sites, where they participate in critical processes such as chlorophyll synthesis, energy transfer, metabolism, and cell division [58], all of which ultimately support and promote flower development [59, 60]. Also, complementary explanation is that the improved flowering resulted from an overall enhancement in the trees’ nutritional status. This would lead to a greater availability of assimilates for developing floral organs, an effect achieved through the strategic application of nano fertilizers during the critical pre bloom period [53]. This interpretation aligns strongly with the recognized importance of the pre bloom differentiation phase in determining the successful development of perfect flowers.

Moreover, the findings of this study provide clear evidence that foliar applications of NPK-NF and CaB-NF during ‘off-year’ season enhanced fruit set and yield in ‘Picual’ olive trees, while simultaneously reducing fruit drop and improving fruit retention (Fig. 5). These results align with a substantial body of literature documenting the influence of plant nutrition on reproductive success in fruit trees. The role of nitrogen and phosphorus in promoting fruit set is well established. Cimato et al. [9] first reported that nitrogen fertilization increases fruit set in olive. Buwalda et al. [61] similarly demonstrated that nitrogen deficiency reduces fruit set in kiwifruit. Haberman et al. [62] recently observed that nitrogen deficiency adversely affects both fruit set and yield. Erel et al. [7, 52] subsequently demonstrated that nitrogen and phosphorus availability significantly influences fruit set in olive. Kumar et al. [63] further illustrated that phosphorus, in combination with nitrogen and potassium, affects fruiting, yield, and fruit quality in guava. Davarpanah et al. [64] found that nano nitrogen applications enhanced fruit yield in pomegranate. Saied [24] reported improvements in mango yield and fruit retention using nano NPK magnesium fertilizers. Gad et al. [59] showed that foliar applications of nano potassium silicate improved final fruit set, yield, and fruit retention in the ‘Ewais’ mango cultivar. Most recently, Abdelrahman et al. [65] reported that nitrogen application influences fruit set, yield, and fruit quality in ‘Valencia’ orange trees. The contribution of boron and calcium to reproductive success is also well documented. Wang JiaWei et al. [66] emphasized that foliar fertilization with boron and calcium is essential for flowering and fruit set in olives. El Hady et al. [54] provided particularly relevant evidence, demonstrating that spraying ‘Kalamata’ and ‘Manzanillo’ olive cultivars in late December with 0.5% chelated calcium increased initial and final fruit set, as well as yield per tree, while decreasing fruit drop. Recently, Esetlili et al. [67] provided evidence that foliar Ca and B treatments increase yield in olive. Also, Muengkaew et al. [68] demonstrated that calcium and boron enhance pollen germination and fruit set in mango. Bibi et al. [69] further reported that foliar applications of boron combined with calcium and potassium improve fruit yield and quality in mango. Regarding nano application, Vishekaii et al. [53] showed that foliar applications of nano boron improved fruit set percentages in olive. The improvements in fruit set and yield observed in the present study can be attributed to the specific physiological roles of the nutrients supplied (Fig. 5). Nitrogen, phosphorus, and potassium provide essential support for plant growth, flower formation, and fruit development [70, 71]. Nitrogen promotes photosynthesis, cell division, and cell elongation, and enhances meristematic activity by contributing to the synthesis of DNA, RNA, amino acids, chlorophyll, plant hormones such as IAA, vitamins, and enzymes [72–74]. Phosphorus plays a crucial role in the formation of nucleic acids, energy compounds including ATP and ADP, and proteins, all of which are essential for cell division and differentiation during flower and fruit development [71]. Potassium is similarly indispensable for various enzymatic activities, sugar transport, and water regulation, processes that directly influence fruit set and subsequent development [75]. The enhancement in initial and final fruit set may also be linked to the specific role of boron, which is critically important for pollen grain germination and pollen tube growth [76]. Furthermore, the reduction in fruit drop (i.e., improved fruit retention) may be attributed to the role of calcium (Fig. 5c). Calcium regulates abscission by modulating hormone signaling and influencing cell wall alterations within the abscission zone [77]. Arseneault et al. [78] demonstrated that calcium can affect the activity of cell wall modifying enzymes, such as cellulases and polygalacturonases, thereby influencing the timing and extent of abscission. Moreover, Xiong et al. [79] showed that calcium specifically interacts with ethylene and abscisic acid hormonal pathways to regulate the abscission process. Furthermore, complementary explanation is that the improvements in fruit yield and retention resulted from enhanced nutrient concentrations in leaves, which significantly affect fruit development and overall tree productivity [59, 80].

As reported, the highest fruit weight was recorded for the control treatment. This observation may be explained by the fact that the control trees exhibited the lowest final fruit set, as presented in Fig. 5, and consequently bore the smallest number of fruits per tree. An inverse relationship between fruit number per tree and individual fruit weight is well documented; as the number of fruits increases, the average weight of each fruit tends to decrease [81–83]. This phenomenon occurs because the tree’s resources, including carbohydrates, water, and nutrients, are distributed among all developing fruits [82]. When a tree carries a heavy fruit load, each fruit receives a smaller share of these limited resources, thereby constraining its potential size and weight [83, 84].

The observed improvements in the nutrient content of olive leaves following the application of NPK-NF and CaB-NF (Fig. 7) are consistent with previous findings. Erel et al. [7] confirmed that the addition of NPK fertilizers to olive trees led to substantial increases in the concentrations of these nutrients in leaves. It should be noted that baseline leaf nutritional status data were not available, representing a limitation of this study. While our experimental design ensured that all trees were uniform in size, shape, and productivity and had been subjected to the same horticultural practices including irrigation and fertilization, we did not collect leaf samples at the start of the experiment to determine initial nutrient concentrations. Such baseline data would have allowed us to more precisely assess the extent to which foliar treatments altered leaf nutrient dynamics over time. However, because the same control treatment was applied across all seasons and all trees received identical background management, the relative differences observed between treated and untreated trees remain valid. Future studies should include baseline leaf nutrient analysis to provide a more complete understanding of treatment effects on tree nutritional status. Hagagg et al. [28] found that applying nano NPK fertilizers at a concentration of 0.2% to ‘Kalamata’ olive trees increased leaf nitrogen, phosphorus, and potassium levels. Mustafa et al. [85] reported that the application of NPK nano fertilizer to ‘Sultani’ fig seedlings resulted in a significant increase in leaf nitrogen, phosphorus, and potassium content. Also, Stojanova et al. [86] also confirmed that the foliar addition of mineral NPK to the ‘Stenlej’ plum variety enhanced the leaf and fruit contents of nitrogen, phosphorus, and potassium. More recently, Al-Karam et al. [87] reported that spraying pear saplings with NPK nano fertilizer led to significant increases in leaf nitrogen, phosphorus, and potassium. Concerning boron and calcium, Davarpanah et al. [88] found that application of nano boron at 6.5 mg/L resulted in the highest leaf boron concentration in pomegranate. Hegazi et al. [89] demonstrated that foliar boron application was significantly effective in increasing boron concentration in olive leaves. Hagagg et al. [90, 91] further indicated that foliar calcium application on ‘Manzanillo’ and ‘Kalamata’ olive trees in December increased leaf mineral content, including nitrogen, phosphorus, potassium, and calcium. The enhanced nutrient uptake observed with nano fertilizers (Fig. 7) can be attributed to their smaller particle size and larger surface area, which improve nutrient uptake efficiency [57]. This characteristic helps explain the increased elemental content in olive leaves (Fig. 7; panels a‒e). It may also be due to the fact that when nitrogen, phosphorus, and potassium are applied, the availability of these elements increases, thereby raising their concentration in leaves through their involvement in biological processes. Nitrogen and phosphorus contribute to the formation of compounds essential for photosynthesis and respiration, while potassium facilitates enzyme formation, further enhancing the availability of these elements within the leaf [72–74]. These combined effects likely led to the observed increase in leaf mineral content. The improvement in fruit nitrogen, phosphorus, and potassium content resulting from the application of nano NPK and nano CaB (Fig. 7f, g, and h) aligns with findings reported by several researchers. Elmer et al. [92] observed that exogenous calcium treatment may increase calcium concentration in peach. Lötze and Theron [93] reported similar findings in ‘Golden Delicious’ apple fruit. Ghani et al. [94] confirmed that calcium application increased calcium concentration in dragon fruit. Ranjbar et al. [95] indicated that spraying apple trees with nano calcium at a concentration of 2.0% enhanced fruit calcium concentration. Also, Morales Sillero et al. [96] confirmed that foliar application of calcium on olive trees increased fruit calcium content. More recently, Saqr et al. [97] demonstrated that spraying date palm with nano macro elements (NPK) at a concentration of 150 mg/L significantly increased fruit nitrogen, phosphorus, and potassium content. The differences in olive fruit nutrient content between the control and treated trees can be attributed to the application of nano fertilizers (Fig. 7; panels f‒j). Owing to their diminutive size, nano fertilizers exhibit enhanced bioavailability, which may augment nutrient uptake efficiency in plants [56, 57, 67]. This can lead to changes in the mineral composition of olive fruit, thereby affecting its quality and yield. Furthermore, the observed differences in vegetative growth characteristics among treatments may be attributed to variations in yield. The control treatment, which produced the lowest yield as shown in Fig. 5, exhibited the highest vegetative growth as presented in Fig. 8. Conversely, T7 (NPK NF at 4 mL/L combined with CaB NF at 2 mL/L) recorded the highest yield alongside the lowest vegetative growth. This inverse relationship between yield and vegetative growth in olive trees can be explained by resource allocation and the resulting competition between reproductive and vegetative processes [98]. High fruit loads create increased demand for resources such as nutrients and water, thereby limiting the availability of these resources for vegetative growth [99].

The observed increase in olive oil content following nano fertilizer application suggests that enhanced foliar nutrition promotes the biochemical pathways responsible for triglyceride synthesis and accumulation in the fruit mesocarp (Table 1). The concurrent decrease in moisture content is a well-documented physiological response during olive ripening, where water is progressively replaced by accumulating lipids [22, 67]. Crucially, while nano fertilization improved oil yield (Table 1), it did not compromise oil quality (Tables 2 and 3, and 4). All measured quality indices including free fatty acids, peroxide value, K232, and K270 remained strictly within the stringent limits set by the International Olive Council for extra virgin olive oil. This reinforces the understanding that while mineral nutrition can significantly influence tree productivity and primary metabolites like oil, it has a limited effect on the hydrolytic and oxidative degradation products that define these key quality parameters [67]. Also, the reduction in phenolic compounds and associated antioxidant activity under higher fertilization rates is a noteworthy finding (Table 3). Phenolic compounds are secondary metabolites critical to olive oil’s nutritional value, sensory characteristics including bitterness and pungency, and oxidative stability [16, 100]. Their decline can be explained by a nutrient-induced shift in plant metabolism (Table 3). Enhanced availability of nutrients, particularly nitrogen, can prioritize primary metabolic processes such as growth and lipid synthesis over secondary metabolic pathways like the phenylpropanoid pathway, which is responsible for phenolic biosynthesis [101, 102]. This inverse relationship between nutrient supply and phenolic accumulation has been similarly reported by Hmmam et al. [22] and Zipori et al. [17]. Moreover, the contrasting increase in carotenoid and chlorophyll pigments with nano fertilization is likely a direct result of improved plant nutritional status (Table 3). An adequate supply of key nutrients, especially nitrogen and magnesium as components of nano NPK, is essential for the synthesis and stability of chloroplast membranes and photosynthetic pigments [103]. This enhanced photosynthetic capacity may, in turn, contribute to the greater oil accumulation observed. Furthermore, the positive impact on fatty acid composition, particularly the increase in oleic acid and the decrease in linoleic acid, represents another qualitative improvement (Table 4). This shift suggests a potential downregulation or inhibition of the enzyme oleate desaturase, which converts oleic acid to linoleic acid [22]. A higher oleic to linoleic acid ratio is a highly desirable trait, as it enhances the oil’s oxidative stability, extends its shelf life, and improves its nutritional profile [16, 18, 67, 104]. These findings corroborate those of Erfani-Moghadam and Zarei [104] and Esetlili et al. [67], who also found that nano nutrient applications can beneficially modulate olive oil fatty acid profiles. The marginal elevation of linolenic acid near the IOC limit in some samples, however, warrants attention to ensure that optimal fertilization rates are not exceeded.

## Conclusion

The present investigation demonstrates that foliar application of NPK and calcium-boron fertilizers constitutes an effective orchard management practice for enhancing the productive performance of ‘Picual’ olive trees. Among the treatments evaluated, T7 (NPK-NF (4 mL/L) + CaB-NF (2 mL/L)) consistently produced the most favorable outcomes across the evaluated parameters during the ‘off-year’ season. This specific combination maximized inflorescence production, flowering density, perfect flower percentage, and both initial and final fruit set while minimizing fruit drop, ultimately yielding the highest ‘off-year’ production among all treatments. The superior reproductive performance observed with this treatment corresponded directly with elevated nitrogen, phosphorus, potassium, and calcium concentrations measured in both leaf tissues and fruit flesh, confirming that improved nutrient availability during critical phenological windows translates into enhanced flowering and fruiting success. Notably, control trees exhibited greater vegetative growth, indicating a preferential allocation of assimilates to shoot development in the absence of nano-fertilizer application. In contrast, nano-fertilized trees demonstrated more balanced resource partitioning, enhancing flowering and fruiting while limiting excessive vegetative vigor. Regarding virgin olive oil quality, all treatments produced oil that satisfied International Olive Council criteria for extra virgin classification, with free fatty acid values and peroxide levels remaining well within established limits. The substantial increase in oil content observed in nano treated fruits compared to control represents an economically significant improvement for olive growers. Interestingly, observations from the ‘on-year’ season revealed that trees treated with nano fertilizers experienced substantially lower alternate bearing severity relative to untreated controls. In conclusion, the evidence presented supports the integration of balanced NPK and calcium-boron foliar nutrition into olive orchard management programs as a means of enhancing reproductive performance, improving oil quality attributes, and addressing the longstanding challenge of alternate bearing in this economically important species.

### Future perspectives

Future work should assess whether adjusting the timing or rate of fertilizer application becomes necessary as the nutrient status of the trees improves. A more mechanistic understanding of how enhanced mineral nutrition influences reproductive development in olive could be gained through advanced molecular approach. Examining the expression patterns of genes involved in flower induction and differentiation in response to varying nutrient regimes may help identify the molecular pathways through which nutrient availability modulates the transition from vegetative to reproductive growth. Equally important is the integration of balanced foliar nutrition with complementary sustainable practices, such as regulated deficit irrigation and organic amendments. Investigating the synergistic effects of these combinations may identify configurations that maximize yield while enhancing resource efficiency. Ultimately, such holistic strategies will be central to developing resilient, environmentally responsible olive production systems that sustain productivity and safeguard ecological integrity for the long term.

## Supplementary Information

Supplementary Material 1.

Supplementary Material 2.

Supplementary Material 3.

## Data Availability

The data generated during and/or analysed during the current study are available from the corresponding author on reasonable request.
