# Supplementary material for: Foliar application of nano-NPK and calcium-boron during the ‘off-year’ season enhances yield, improves oil quality, and mitigates biennial bearing in ‘Picual’ olive cultivar
Source: BMC Plant Biol. 2026 May 25;26:913. doi: 10.1186/s12870-026-08984-y (PMC13200473; doi:10.1186/s12870-026-08984-y)
Supplement: Supplementary file 3 — Supplementary Material 3. [file 12870_2026_8984_MOESM3_ESM.docx]

**Supplementary Table S1. Season × Treatment means for Leaf nitrogen (%)**

| **Treatment** | **1^st^ season Mean ± SE** | **DMRT**  **(S1)** | **2^nd^ season**  **Mean ± SE** | **DMRT**  **(S2)** |
| --- | --- | --- | --- | --- |
| T0 | 2.620 ± 0.095 | cd | 2.113 ± 0.007 | b |
| T1 | 2.756 ± 0.058 | cd | 2.176 ± 0.038 | ab |
| T2 | 2.586 ± 0.058 | d | 2.280 ± 0.102 | ab |
| T3 | 2.590 ± 0.085 | d | 2.203 ± 0.042 | ab |
| T4 | 2.800 ± 0.076 | bc | 2.293 ± 0.065 | ab |
| T5 | 2.800 ± 0.108 | bc | 2.253 ± 0.038 | ab |
| T6 | 2.780 ± 0.104 | bc | 2.213 ± 0.026 | ab |
| T7 | 3.016 ± 0.191 | a | 2.263 ± 0.046 | ab |
| T8 | 2.953 ± 0.158 | ab | 2.260 ± 0.081 | ab |
| T9 | 2.806 ± 0.052 | bc | 2.326 ± 0.052 | a |
| **Season mean** | **2.770 ± 0.044 a** |  | **2.238 ± 0.023 b** |  |

**Supplementary Table S2. Season × Treatment means for Leaf calcium (%)**

| **Treatment** | **1^st^ season Mean ± SE** | **DMRT**  **(S1)** | **2^nd^ season**  **Mean ± SE** | **DMRT**  **(S2)** |
| --- | --- | --- | --- | --- |
| Control | 1.973 ± 0.046 | e | 1.820 ± 0.064 | e |
| T1 | 2.296 ± 0.026 | d | 2.103 ± 0.073 | d |
| T2 | 2.333 ± 0.069 | d | 2.280 ± 0.061 | ab |
| T3 | 2.413 ± 0.042 | bc | 2.333 ± 0.034 | a |
| T4 | 2.346 ± 0.029 | cd | 2.186 ± 0.085 | bcd |
| T5 | 2.446 ± 0.024 | ab | 2.220 ± 0.110 | abc |
| T6 | 2.430 ± 0.038 | ab | 2.330 ± 0.036 | a |
| T7 | 2.476 ± 0.028 | ab | 2.110 ± 0.117 | cd |
| T8 | 2.430 ± 0.044 | ab | 2.250 ± 0.051 | ab |
| T9 | 2.496 ± 0.064 | a | 2.323 ± 0.071 | a |
| **Season mean** | **2.363 ± 0.030 a** |  | **2.195 ± 0.032 b** |  |

**Supplementary Table S3. Season × Treatment means for Off-year yield (ton/acre)**

| **Treatment** | **1^st^ season Mean ± SE** | **DMRT**  **(S1)** | **2^nd^ season**  **Mean ± SE** | **DMRT**  **(S2)** |
| --- | --- | --- | --- | --- |
| Control | 0.121 ± 0.007 | h | 0.101 ± 0.013 | g |
| T1 | 0.475 ± 0.046 | b | 0.372 ± 0.020 | b |
| T2 | 0.306 ± 0.019 | de | 0.265 ± 0.015 | d |
| T3 | 0.187 ± 0.012 | g | 0.158 ± 0.009 | f |
| T4 | 0.510 ± 0.033 | b | 0.420 ± 0.032 | a |
| T5 | 0.341 ± 0.028 | d | 0.276 ± 0.026 | cd |
| T6 | 0.232 ± 0.028 | fg | 0.187 ± 0.026 | ef |
| T7 | 0.578 ± 0.056 | a | 0.434 ± 0.022 | a |
| T8 | 0.411 ± 0.034 | c | 0.317 ± 0.026 | c |
| T9 | 0.255 ± 0.020 | ef | 0.214 ± 0.015 | e |
| **Season mean** | **0.341 ± 0.049 a** |  | **0.274 ± 0.033 b** |  |
